# NAT10/ac4C/JunB facilitates TNBC malignant progression and immunosuppression by driving glycolysis addiction

**DOI:** 10.1186/s13046-024-03200-x

**Published:** 2024-10-04

**Authors:** Guozheng Li, Xin Ma, Shiyao Sui, Yihai Chen, Hui Li, Lei Liu, Xin Zhang, Lei Zhang, Yi Hao, Zihan Yang, Shuai Yang, Xu He, Qin Wang, Weiyang Tao, Shouping Xu

**Affiliations:** 1 Department of Breast Surgery, Harbin Medical University Cancer Hospital, Harbin, 150040 China; 2 Key Laboratory of Tumor Biotherapy of Heilongjiang Province, Harbin Medical University Cancer Hospital, Harbin, 150081 China; 3 Department of Breast Surgery, The First Affiliated Hospital of Harbin Medical University, Harbin, 150001 China; 4 National Center for Nanoscience and Technology, Beijing, 100190 China; 5 Weihan Yu Academy, Harbin Medical University, Harbin, 150086 China

**Keywords:** Triple negative breast cancer, N4-acetylcytidine, NAT10/ac4C/JunB axis, Glycolysis, Immunosuppression, CTLA-4

## Abstract

**Background:**

N4-Acetylcytidine (ac4C), a highly conserved post-transcriptional mechanism, plays a pivotal role in RNA modification and tumor progression. However, the molecular mechanism by which ac4C modification mediates tumor immunosuppression remains elusive in triple-negative breast cancer (TNBC).

**Methods:**

NAT10 expression was analyzed in TNBC samples in the level of mRNA and protein, and compared with the corresponding normal tissues. ac4C modification levels also measured in the TNBC samples. The effects of NAT10 on immune microenvironment and tumor metabolism were investigated. NAT10-mediated ac4C and its downstream regulatory mechanisms were determined in vitro and in vivo. The combination therapy of targeting NAT10 in TNBC was further explored.

**Results:**

The results revealed that the loss of NAT10 inhibited TNBC development and promoted T cell activation. Mechanistically, NAT10 upregulated JunB expression by increasing ac4C modification levels on its mRNA. Moreover, JunB further up-regulated LDHA expression and facilitated glycolysis. By deeply digging, remodelin, a NAT10 inhibitor, elevated the surface expression of CTLA-4 on T cells. The combination of remodelin and CTLA-4 mAb can further activate T cells and inhibite tumor progression.

**Conclusion:**

Taken together, our study demonstrated that the NAT10-ac4C-JunB-LDHA pathway increases glycolysis levels and creates an immunosuppressive tumor microenvironment (TME). Consequently, targeting this pathway may assist in the identification of novel therapeutic strategies to improve the efficacy of cancer immunotherapy.

**Supplementary Information:**

The online version contains supplementary material available at 10.1186/s13046-024-03200-x.

## Background

As is well documented, breast cancer is the most prevalent cancer among females, with the number of new cases increasing from 24.2% in 2018 to 30% in 2021 [1]. TNBC accounts for approximately 15%-20% of these cases [2]. It is characterized by a significantly shorter overall survival rate than other types of breast cancer, high metastasis rates, and chemotherapy resistance, resulting in a lack of effective therapeutic strategies for its treatment [3, 4]. Moreover, in light of anti-human epidermal growth factor therapy and endocrine therapy for other types of breast cancer, effective treatment options for TNBC are lacking.

Recently, significant advances have been made in tumor immunotherapy, and therapeutic monoclonal antibodies targeting programmed cell death protein 1 (PD-1)/programmed cell death 1 ligand 1 (PD-L1) have been extensively used for the treatment of TNBC [5]. The PD-1 monoclonal antibody pembrolizumab has achieved satisfactory therapeutic effects in the first-line treatment of metastatic TNBC [6]*.* Likewise, atezolizumab, a monoclonal antibody targeting PD-L1, was approved in 2018 in combination with Abraxane for the treatment of unresectable locally advanced or metastatic TNBC and markedly improved patient survival outcomes in patients with TNBC [7]. Furthermore, ipilimumab, an antibody against cytotoxic T lymphocyte associate protein-4 (CTLA-4) ipilimumab, was approved in 2011. Clinical phase-III trials investigating the efficacy and safety of ipilimumab are conducted in advanced melanoma patients revealing prolonged overall survival [8, 9]. Further, it has also been applied in the treatment of renal cell carcinoma [10], lung cancer [11], and mesothelioma [12].

Despite immunotherapy with immune checkpoint inhibitors (ICBs) demonstrating clinical efficacy in tumor patients, its effectiveness in the treatment of TNBC remains suboptimal and fails to adequately meet clinical needs [13]. Multiple mechanisms lead to immune escape in TNBC, resulting in poor efficacy of immunotherapy for TNBC, compared to melanoma, renal, and lung cancers [14, 15]. Several mechanisms can promote immune evasion of breast cancer, resulting in a cold tumor phenotype. Expression of inhibitory costimulatory molecules (PD-L1, CTLA-4, and LAG-3) [16]; Infiltration of various immunosuppressive cells (CD4^+^ Th2 cells, regulatory T cell, myeloid-derived suppressor cells, and M2-like macrophage) [17]; Secretion of inhibitory factors (lactate, interleukin-10 (IL-10), and TGF-β) [18–20]; High metabolic activity of tumor cells which consume large amounts of nutrients such as glucose, thereby suppressing the proliferation and activation of immune cells [21]. Therefore, there is a pressing need to identify novel therapeutic strategies and targets to ameliorate the immunosuppressive TME and achieve favorable results.

NAT10 was first reported in 2003, and it is a nucleolar protein comprising 872 amino acids and containing an acetyltransferase domain and a lysine-rich C-terminus [22, 23]. In addition to catalyzing ac4C modification of tRNA and rRNA, NAT10 also contributes to the ac4C modification of mRNA, thus promoting mRNA stability and translation [24, 25]. The catalyzing process depends on the acetyl from Acetyl CoA, and the energy from adenosine triphosphate (ATP) hydrolysis [26]. However, it is unclear whether ac4C modifications on various RNAs can be removed through deacetylation. Compared with m6A methylation, the abundance of ac4C RNA modification is much lower, making its discovery more challenging [27].

Notably, the oncogenic effects of NAT10 promote the progression of various tumors. LINC00623 can bind to NAT10 and prevent its ubiquitination-dependent degradation by recruiting the deubiquitinase USP39, thereby driving the proliferation and metastasis of pancreatic ductal adenocarcinoma [28]. An earlier study reported that NAT10 promotes gastric cancer metastasis and is correlated with a poor prognosis [29]. High expression of NAT10 confers resistance to platinum-based drugs, DNA-damaging chemotherapy, and radiotherapy in patients with breast cancer [30, 31]. Remodelin, a NAT10 inhibitor, mediates mitochondrial fatty acid lipid accumulation and metabolism, thereby decreasing the levels of total triglycerides and cholesterol in cancer cells [32]. Nonetheless, its immunoregulatory function in cancer remains underexplored.

This study exposed that NAT10 promotes TNBC progression. Mass cytometry by time of flight (CyTOF) and in vitro experiments demonstrated that the loss of NAT10 promoted T cell activation and remodeled the TME by down-regulating JunB expression, thus reducing the TNBC glycolysis level. Moreover, remodelin treatment up-regulated the expression of CTLA-4 on the T cell surface. Finally, the combination of remodelin and CTLA-4 mAb inhibited tumor progression and concurrently promoted immune cell infiltration. Collectively, this study established the regulatory role of NAT10 in immune escape mediated by JunB in TNBC, positioning it as a potential target for TNBC immune therapy.

## Methods

### Breast-tissue specimens and clinical assessments

Patients at the Harbin Medical University Cancer Center (HMUCC) with a histological diagnosis of breast cancer who had not received chemotherapy or radiotherapy before surgical resection were considered eligible for recruitment to this study. RNA was extracted from breast cancer and normal control tissues, which was stored at − 80 °C immediately after resection. Paraffin-embedded tissue sections were produced from tissue samples stored in 4% formaldehyde at 4 °C immediately after resection. This study was approved by and conformed to the clinical research guidelines of the Research Ethics Committee of Harbin Medical University Cancer Hospital (KY2022-43). Written informed consent was obtained from all patients.

### Analysis of NAT10 expression in TCGA and GEO database

Breast cancer mRNA NAT10 expression levels were retrieved from the TCGA database (https://portal.gdc.cancer.gov/), and TNBC patients were screened according to Fudan typing (FUTURE). Scatter plots of expression levels were produced for each group. TNBC immunotherapy-related gene expression data were extracted from the Gene Expression Omnibus (GEO) database (https://www.ncbi.nlm.nih.gov/gds/). A dataset (GSE124821) was used for our study, and the experiment type was microarray expression profiling. The expression of the NAT10 gene was extracted, and grouped violin plots were produced. All gene expression data were log2-transformed. Results were visualized using the R software package ggplot.

### Cell culturing and transfection

Breast cancer cell lines (4T1, BT549, MDA-MB-231, and MDA-MB-468), Jurkat, and THP-1 cells were obtained from the Chinese Academy of Sciences Cell Bank and Cellbio. All cell lines were cultured under standard conditions, as specified by the suppliers, using a culture medium supplemented with 100 U/mL penicillin, 100 mg/mL streptomycin, and 10% fetal bovine serum (FBS) at 37 °C with 5% CO2. Cells were seeded in six-well culture plates, at a density of approximately 70%, and were transfected using JetPrime for 48 h (#114–15, Polyplus, Germany) (2 ml culture medium + 4 μl jet + 0.2 nM siRNA). The concentration of remodelin was 50 μM.

### Wound-healing assays

4T1 cells, BT549, and MDA-MB-468 cells (transfected for 36 h or 70 µM remodelin treated for 24h) were seeded in six-well culture plates on RPMI-1640 or DMEM medium containing 5% FBS and were cultivated to a sub-confluent state. A wound was scratched at the bottom of the plate using a 10 µL pipette tip. Cell migration was observed and calculated at the indicated times (0h, 12h, 24h, 36h, 48h), and the size of the remaining wound was measured using an inverted light microscope.

### Invasion assays

0.8–1 × 10^5^ 4T1 cells, BT 549, and MDA-MB-468 cells (transfected for 36 h or 70 µM remodelin treated for 24h) in serum-free conditioned medium were seeded into a BRAND Insert with Matrigel (#BR782806, Sigma-Aldrich, USA). A complete medium containing 20% FBS was added to the lower chamber. After incubation at 37 °C 48 h, cells that did not migrate to the lower chamber were removed, and the invaded cells were fixed with 4% methanol for 30 min and were stained using crystal violet for 30 min. Cells were imaged and counted under a light microscope.

### Cell-viability assays

3–5 × 10^3^ 4T1 cells, BT 549, and MDA-MB-468 cells (transfected for 36 h or 70 µM remodelin treated for 24h) were seeded in 96-well culture plates with 200 μl of the medium. Six wells were plated on the same cells as the replicates. Cell proliferation was observed and calculated at the indicated times (0h, 24h, 48h, 72h). Cell Counting Kit-8 (CCK-8) (#MA0218, Kumamoto, Japan) was added to 10% at 37 °C for 1 h before any measurements. The absorbance of each cell suspension was measured at 450 nm wavelength using a microplate reader.

### THP-1 monocytic cell cultures

We selected the human monocytic leukemia cell line THP-1 (Chinese Academy of Sciences, China), which showed similar characteristics to human macrophage after differentiation. Then, we employed a differentiation and functional polarization protocol that was shown to be effective for THP-1 cells [33, 34]. THP-1 cells were stimulated with phorbol 12-myristate 13-acetate (PMA, 100 ng/ml) at 37°C for 48 h. Then, differentiated THP-1 cells were treated with either lipopolysaccharide (LPS, 100 ng/ml) and IFN-γ (20 ng/ml) for 48 h or IL-4 (20 ng/ml) and IL-13 (20 ng/ml) for 48 h to obtain M1-like and M2-like phenotypes, respectively. Cells were treated with remodelin for 24–48 h after which culture supernatants were analyzed for cytokines. Thereafter, cells were collected and analyzed by flow cytometry.

### Human primary T cell cultures

The peripheral venous blood was collected from healthy volunteers in a heparin anticoagulant tube. Ficoll lymphocyte separation solution (#LTS1077, tbdscience, China) was added to dilute the blood. The PBMC isolation from blood was done using gradient density centrifugation and then counted. The 10 mm dishes were pre-coated with 1 μg/mL anti-CD3 (#16–0037-81, clone OKT3, eBioscience, USA) in PBS at 37°C for 2 h. Next, primary T cells were added in 10^6^ cells/mL suspended in RPMI-1640 which contained 10% FBS, 2 μg/mL anti-CD28(#16–0289-81, clone CD28.2, eBioscience, USA), 200U/mL IL-2, 1% L-glutamine,0.1% β-mercaptoethanol. After three or four days, cells were harvested for experiments.

### In vitro* CTLA-4 cycling*

Human primary T cell was cultured in the presence of anti-CD3 anti-CD28 activation. Then T cell was treated with remodelin for 24–36 h. Staining was carried out at 4 °C to label surface CTLA-4; at 37 °C for 2 h to identify cycling CTLA-4; or after cell fixation and permeabilization to stain the total CTLA-4 pool.

### Qrt-PCR

Total RNA was extracted from cells and tissue samples using TRIzol reagent (#269,201, Invitrogen, USA) according to the manufacturer’s instructions, and RNA was reverse-transcribed into cDNA using a High-Capacity cDNA Reverse Transcription Kit (#4,368,814, Applied Biosystems, USA). mRNA expression was quantified by real-time PCR using an SYBR Green PCR Master Mix Kit (#4,913,914,001, Applied Biosystems) with gene-specific primers. β-actin was used as internal control, and qRT-PCR was performed on a 7500 FAST Real-time PCR System (Applied Biosystems). The results were normalized to β-actin expression levels using the 2–ΔΔCt method. The primer sequences of interest are shown in Supplementary Data S6.

### RNA immunoprecipitation (RIP)

The RNA immunoprecipitation (RIP) assay was performed according to the protocol of the Magna RIP Kit (Millipore, USA). Briefly, 5 μg NAT10 or FLAG antibodies and 50μl magnetic beads were well mixed and incubated with cell lysates. Then, RNAs were extracted after the removal of proteins. Followed by qPCR, the expression of genes was normalized to input.

### RNA pulldown

The cells were transfected with JunB overexpression plasmid (36 h, 500ng/ml). Using biotin labeling to design and synthesize probes based on JunB mRNA. The cell lysate was incubated with beads at 4 °C overnight, which were coated with biotin-tagged JunB. After washing with wash buffer, add elution buffer to separate the RNA–protein complex from the beads. Loading buffer was used to separate the protein and subjected to western blot.

### Seahorse xf96 respirometry

3 × 10^4^ per well BT549 (NAT10 knockdown or JunB overexpression) cells were seeded in the XF96 plate and stabilized overnight. The extracellular acidification rate (ECAR) was measured by the XF96 extracellular flux analyzer with glucose stress fuel flex test kits (Agilent). Measurements of ECAR were performed according to the manufacturer’s instructions. The results were analyzed using Wave software (Seahorse/Agilent).

### Acrip-qpcr assay

The ac4C immunoprecipitation (acRIP) procedure was performed according to instructions issued by the manufacturer using a GenSeq ac4C RIP Kit (#GS-ET-005, CloudSeq Biotech, China). In brief, 100 μg total RNA was isolated from pretreated cells and was randomly fragmented to a size of 200 nucleotides. RNA samples were then immunoprecipitated using magnetic beads pre-coated with anti-ac4C antibody. The ac4C-modified RNA fragments were eluted with nuclease-free water. Enriched ac4C-modified mRNA was then detected through qRT-PCR.

### Chip-qpcr assay

Chromatin-immunoprecipitation (ChIP) assays were performed using a ChIP Assay Kit (#P2078, Beyotime, Shanghai, China), following the manufacturer’s instructions. cells were cross-linked with formaldehyde and sonicated to an average length of 200–1000 bp. The sheared chromatin was immunoprecipitated at 4 ℃ overnight using an anti-JunB antibody (#GTX116011, GeneTex); IgG (BD Biosciences, San Diego, CA) served as the negative control. The precipitated DNA was amplified by RT-PCR.

### RNA decay assay

TNBC cells were treated with vehicle (DMSO) or remodelin and were transfected with siNC or siNAT10. Then TNBC cells were seeded in 12-well plates. Actinomycin D was added into each well at a final concentration of 2 µg/mL. The cells were collected after 0, 30, 60, and 90 min, respectively. Total RNA was isolated and subjected to qRT-PCR to quantify the relative expression of JunB. β-actin was used as an internal control.

### Ac4c dot blot

Total RNA was heated to 95 ℃ for 3 min and was placed on ice, and loaded onto Hybond-N + membranes. Membranes were crosslinked at 150 mJ/cm^2^ in a UV254 nm Stratalinker 2400 (Stratagene, USA), and were blocked with 5% nonfat milk in 0.1% Tween 20 PBS (PBST) for 1 h at room temperature (RT) followed by incubation with an anti-ac4C antibody (#ab252215, Abcam, USA) in PBST (1:250) at 4 ℃ overnight. The membranes were then washed three times using PBST, incubated with an HRP-conjugated secondary anti-rabbit IgG in PBST (1:1000) at RT for 1 h, and washed three times with PBST. The ac4C modification was visualized using an enhanced chemiluminescence detection system (Western Lightning, Perkin Elmer, Norwalk, CT, USA) and the total RNA was visualized by methylene blue.

### Western blot

Cells were lysed with 10 mM Tris–HCl (pH 7.4), 150 mM NaCl, 5 mM NaF, 10 mM DTT, 5% glycerol, 5000 U/ml proteinase inhibitors, and 2% SDS. Protein concentrations were measured using a protein assay kit (#5,000,001, Bio-Rad, Richmond, USA); equal amounts of protein were separated using SDS-PAGE and were then transferred onto polyvinylidene fluoride membranes blocked with 5% skim milk in TBST for 1 h at RT. Then, the membranes were incubated overnight at 4 ℃ with the primary antibody diluted in TBST. After washing three times using TBST, the membranes were incubated with HRP-labeled secondary antibodies for 1 h. After washing with TBST, protein bands were visualized using an enhanced chemiluminescence detection system (Western Lightning, Perkin Elmer).

### Enzyme-linked immunosorbent assay (ELISA)

Cells were seeded in a six-well plate. Cells were treated with vehicle (DMSO) or remodelin and were transfected with siNC or siNAT10. Then supernatants were collected to measure the total levels of several cytokines using respective human ELISA kits according to the manufacturer’s instructions. The plates were read using a microplate reader at 450 nm wavelength.

### Immunohistochemistry and multiplex immunofluorescence staining

Tumor tissues were fixed, embedded, and sectioned (4 μm thickness), followed by backing for 2 h at 60 °C. The slides were then deparaffinized in xylene and rehydrated in gradient ethanol. First, antigen retrieval of slides was performed with citrate buffer (pH 6.0) or EDTA (pH 8.0), using a steamer (95–100 ℃) for 10 min. The slides were incubated in 3% hydrogen peroxide for 15 min to block endogenous peroxidase activity followed by 10% BSA incubation for 1 h at RT. The first primary antibody was applied for 4 ℃ overnight followed by washing three times using PBS. The slides were incubated with appropriate secondary universal immuno-peroxidase polymer, anti-rabbit, or anti-mouse antibody for 30 min, followed by washing thrice. Detection was performed using liquid DAB + (#9018, Golden Bridge, China) and counterstaining with Carazzi’s hematoxylin (#BL702A, Biosharp, China). The stained sections were independently analyzed by two pathologists.

Multiplex immunofluorescence staining was performed by using the four-color multiple fluorescent immunohistochemical staining kit (#abs50012, Absin, China) based on the tyramide signal amplification (TSA) technique, according to the manufacturer’s manual. As described above, after performing antigen retrieval and blocking, we sequentially incubated primary antibodies for 30 to 60 min at 37 ℃, followed by incubation of HRP-conjugated secondary antibody and TSA with Opal. Lastly, sections were counterstained using DAPI and were mounted in glycerol and gelatin mounting medium. Antibody information is listed in Supplementary Data S1. Tissue sections were imaged using a Nikon A1 scanning confocal microscope. Confocal images were captured and the image data were collected using NIS Elements (Nikon, V4.50.00).

### Animal experiment

The animal experiment was approved by the Ethics Committee of the National Center for Nanoscience and Technology (NCNST21-2203–0610). Six-to-eight-week-old female Balb/c mice were obtained from the Beijing Vital River Laboratory Animal Technology Company. Approximately 5 × 10^4^ 4T1 cells in 50 μl of serum-free RPMI1640 medium and 50 μl Matrigel matrix were injected directly into the right mammary fat pad. For in vivo 4T1 tumor-growth experiments, remodelin was administered at a dosage of 3 mg/kg by intraperitoneal injection every four days. Anti-CTLA-4 antibody (#BP0164, BioXCell, USA) was injected intraperitoand and nearly administered at a dosage of 250 µg. Tumor growth was measured once every 2 days using calipers, and tumor volume was calculated as 1/2 (length × width^2^).

### Cytokine panel

Blood was collected after the animal experiments. The samples were centrifuged at 3,000 × *g* at 4 ℃ for 20 min; serum was collected and stored at − 80℃ until use. Serum samples were processed by Univ Bioscience using the Millipore MILLIPLEX MAP Mouse Cytokine/Chemokine 32-Plex panel (#MCYTMAG 70K PX32). Samples were examined according to the manufacturer’s protocol, as follows: serum was thawed and diluted to one-part serum to one-part assay butter on the day of the assay. Fifty microliters sample or standards was plated with 50 µl assay buffer, and 50 µl beads in each well, which were then sealed and agitated for 30 min at RT. The plate was washed three times, then 25 µl of detection antibodies were added to each well and the plate was sealed, covered with foil, and agitated for 30 min at RT. Fifty microliters of streptavidin–phycoerythrin were added to each well. The plate was sealed, covered with foil, and agitated for 10 min at RT. The plate was washed three times, then 125 µl of assay buffer was added to each well, and beads were resuspended on a plate shaker for 30 s. Plates were read on a Luminex 200.

### RNA-sequencing (RNA-seq)

BT549 was transfected with siNC or siNAT10 for 48 h, and total RNA was sent to Majorbio Bio-pharm Biotechnology Co. Ltd. (Shanghai, China) for the construction of libraries. Small RNA libraries were constructed using the Illumina TruSeq Small RNA Kit (Illumina, USA), and strand-specific libraries were constructed using the Illumina TruseqTM RNA sample prep Kit method. RNA-sequencing was performed on an Illumina Novaseq 6000/HiSeq X ten platform (Illumina), according to the manufacturer’s instructions.

### Flow cytometry analysis

Cell surface markers of CD8^+^T, CD4^+^T, macrophage, and cytokines (IL-2, TNF-α, and IFN-γ) assessment was performed by immunofluorescence staining followed by flow cytometry analysis. In brief, cells were collected at 800 × *g* for 5 min and were washed with, and suspended in FACS buffer (#00–4222-26, Thermo Fisher Scientific, USA) (1.0 × 10^6^ cells/100 μl), and all steps were performed in the dark unless stated otherwise. Then cells were incubated with fluorochrome-conjugated anti-CD3, anti-CD4, anti-CD8, anti-CD86, anti- CX3CR1, and anti- MHCII antibodies at 4 °C for 30 min.

For measurement of intracellular CD206, IL-2, TNF-α, and IFN-γ, cells were stimulated with PMA (20 ng/mL), ionomycin (1 ug/mL), and brefeldin A (10 ug/mL) for 4–6 h. And then the cells were washed using FACS buffer, fixed, and permeabilized with intracellular staining fixation buffer (#420,801, BioLegend, USA) at RT for 20 min and intracellular staining permeabilization wash buffer (10X) (#421,002, BioLegend) at 4 ℃ for 40 min followed by staining with fluorochrome-conjugated anti-IL-2, anti-TNF-α, anti-IFN-γ antibodies at RT for 30 min. After incubation, cells were washed with intracellular staining permeabilization wash buffer and FACS buffer and were then suspended in FACS buffer. The specificity of the antibodies was verified by staining with respective isotype control antibodies and FMOs. The samples were analyzed immediately or within 24 h using BD canton II (Thermo Fisher Scientific). Data evaluation was performed using the FlowJo software.

### Time-of-flight mass cytometry (cytof)

Mass cytometry was performed by PLTTech Inc. (Hangzhou, China). Cells isolated from mouse breast cancer tissues (control group and remodelin treated group) were incubated with a surface antibody mix panel and were stained overnight with the DNA Intercalator-Ir (FLUIDIGM, South San Francisco, CA) to differentiate live cells from debris. After fixation and permeabilization, cells were stained using intracellular/nuclear antibodies. The immuno-labeled samples were then barcoded using a unique barcode isotope combination for 30 min, re-suspended in deionized water, and subjected to a CyTOF instrument (Helios, FLUIDIGM). The antibodies used in the assay are listed in the Supplementary Data S1. Following density clustering, the immune cells were identified via t-distributed stochastic neighbor embedding (t-SNE).

### *NAD* + */NADH assay and ATP assay*

TNBC cells were treated with vehicle (DMSO) or remodelin, and transfected with siNC or siNAT10 for 48 h. Then the lysate provided with the kits was used to collect the intracellular fluid for subsequent assays. NAD + , NADH, and total NAD (NADt) (NAD +  + NADH = NADt) were measured using a NAD + /NADH assay kit (#S0179, Beyotime, China) according to the manufacturer’s instructions. ATP was detected using a kit from the Nanjing Jiancheng Bioengineering Institute (#A095-1–1, Nanjing Jiancheng, China).

### Statistical analyses

Statistical analyses were conducted using GraphPad Prism (GraphPad Software, USA) using the Student’s t-test and ANOVA test. Spearman’s correlation coefficients were calculated for correlation analyses. All experiments were conducted using three replicates. Statistical significance was defined as *P* < 0.05.

## Results

### NAT10 plays a potential oncogenic role in TNBC.

**Fig. 1:**
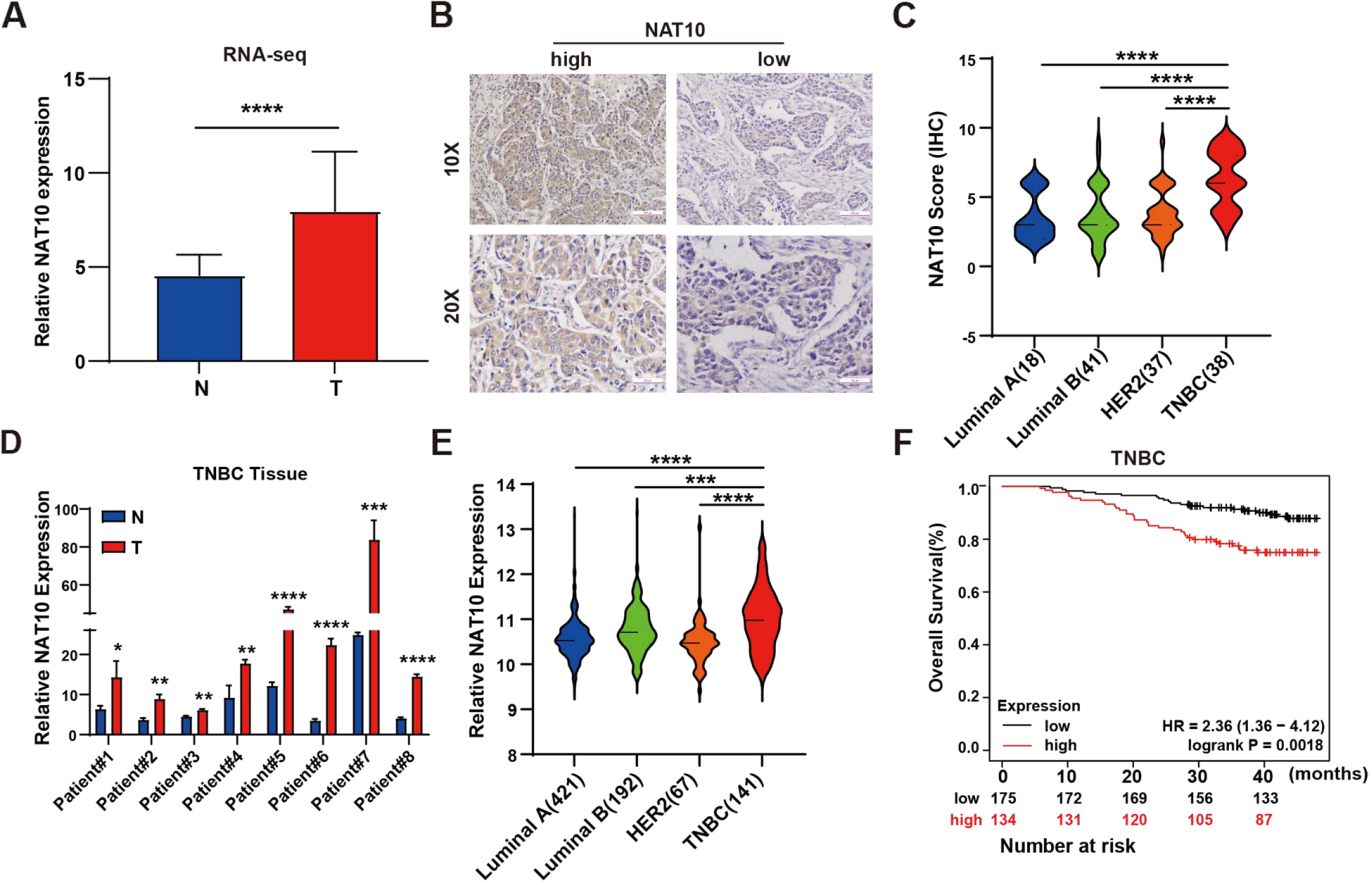
NAT10 Plays a Potential Oncogenic Role in TNBC. **A** RNA-seq detected NAT10 expression in 15 normal tissues (N) and 15 tumor tissues (T) of breast cancer. **B**, **C** IHC detected NAT10 protein expression from 134 breast cancer patients in the HMUCC cohort, including 18 Luminal A subtypes, 41 Luminal B subtypes, 37 HER2 subtypes, and 38 TNBC subtypes. **D** qRT-PCR detected NAT10 expression in normal tissues (N) and tumor tissues (T) of TNBC. **E** NAT10 expression in four molecular types of 821 breast cancers in the TCGA database, including 421 Luminal A subtypes, 192 Luminal B subtypes, 67 HER2 subtypes, and 141 TNBC subtypes. **F** Kaplan–Meier analysis of overall survival of TNBC patients according to NAT10 expression by using KM plotter (https://kmplot.com/analysis/index.php?p = service&cancer = breast_rnaseq_gse96058). The data are shown as the means ± SDs; *P < 0.05, **P < 0.01, ***P < 0.001, ****P < 0.0001

**Fig.2:**
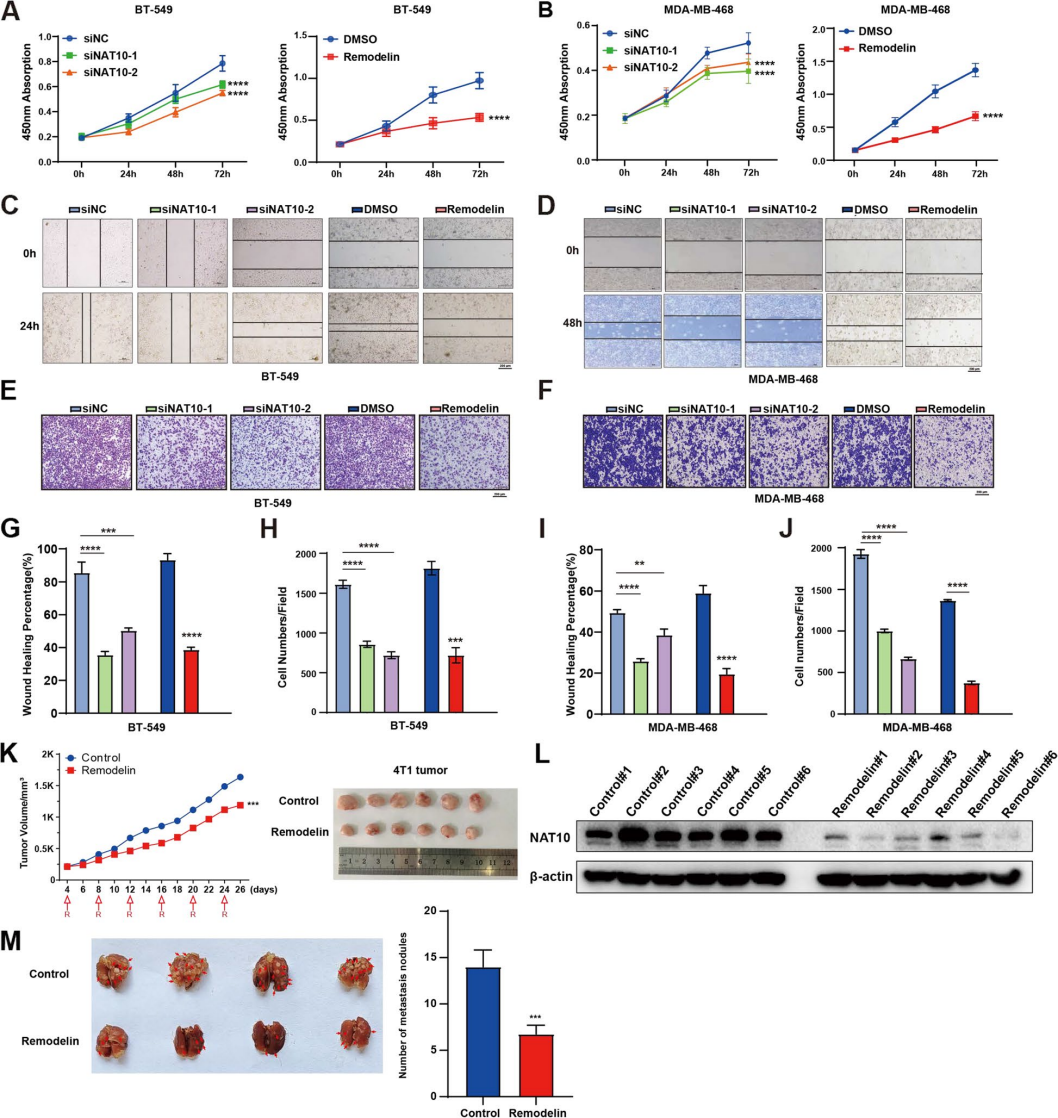
NAT10 facilitates the TNBC tumorigenesis. **A**, **B** The loss of NAT10 inhibits the proliferation. **C**-**J** The loss of NAT10 inhibits invasion and migration. **K** 4T1 tumor volume was measured after remodelin treatment. **L** Detect the efficiency of NAT10 knockdown in the mice tissues by western blot. **M** Lung metastasis nodules of the 4T1 tumor were measured after remodelin treatment. The data are shown as the means ± SDs; ***P < 0.001, ****P < 0.0001

RNA-sequencing analysis from 15 pairs of breast cancer tissues demonstrated that NAT10 was highly expressed in breast cancer tissues (Fig. 1A and Supplementary Fig. S1A). Likewise, immunohistochemistry (IHC) analysis of NAT10 expression in 134 breast cancer patients demonstrated that NAT10 is highly expressed in TNBC (Fig. 1B, C). Furthermore, its mRNA expression was also high in TNBC tissues (Fig. 1D), consistent with the results of TCGA datasets analysis (Fig. 1E). The level of ac4C modifications was significantly higher in TNBC tissues compared to adjacent tissues, except for patients #6 and #10 (Supplementary Fig. S1B). NAT10 also expressed high levels in TNBC cell lines (Supplementary Fig. S1C). As anticipated, high NAT10 expression was associated with a markedly poorer prognosis in TNBC patients (Fig. 1F). These results conjointly indicated that NAT10 facilitated the progression of breast cancer by regulating ac4C modification in TNBC. To explore the role of NAT10 in TNBC progression. siRNA and remodelin were used to knock down NAT10 (NAT10-Kd) expression (Supplementary Fig. S1D-F). Quantification of the ac4C level in tumor cells showed that ac4C modification on mRNA decreased in the NAT10-Kd cells (Supplementary Fig. S1G). In addition, the in vitro assays showed that NAT10 promoted the proliferative and invasive abilities of TNBC cells (Fig. 2A-J and Supplementary Fig. S2, 3). In 4T1 cell tumor-bearing mice, treatment with remodelin significantly inhibited tumor growth and lung metastasis (Fig. 2K-M).

### NAT10 function is required for immunosuppressive TME in TNBC

To identify the mechanism by which NAT10 regulates TNBC progression, a TNBC dataset based on the Fudan subtypes [35] was retrieved from the TCGA database and analyzed, exposing that NAT10 expression was significantly higher in the basal-like immune suppressed (BLIS) subtype compared to that in other subtypes (Supplementary Fig. S4A). Next, the TIMER2 (Tumor Immune Estimation Resource 2) database was employed for further analysis, identifying that NAT10 expression was positively correlated with M2-like macrophages, CD4 + Th2 cells, and myeloid-derived suppressor cells and negatively correlated with CD4 + Th1 cells (Supplementary Fig. S4B). Interestingly, the results of TIMER2 analysis implied that CD4^+^T cell, CD8^+^T cell, and M1-like macrophage were associated with a good prognosis in breast cancer, whereas M2-like macrophage was associated with a poorer prognosis (Supplementary Fig. S4C). These findings strongly suggest that NAT10 may facilitate breast cancer progression by inhibiting immune cell infiltration or activation.

**Fig. 3:**
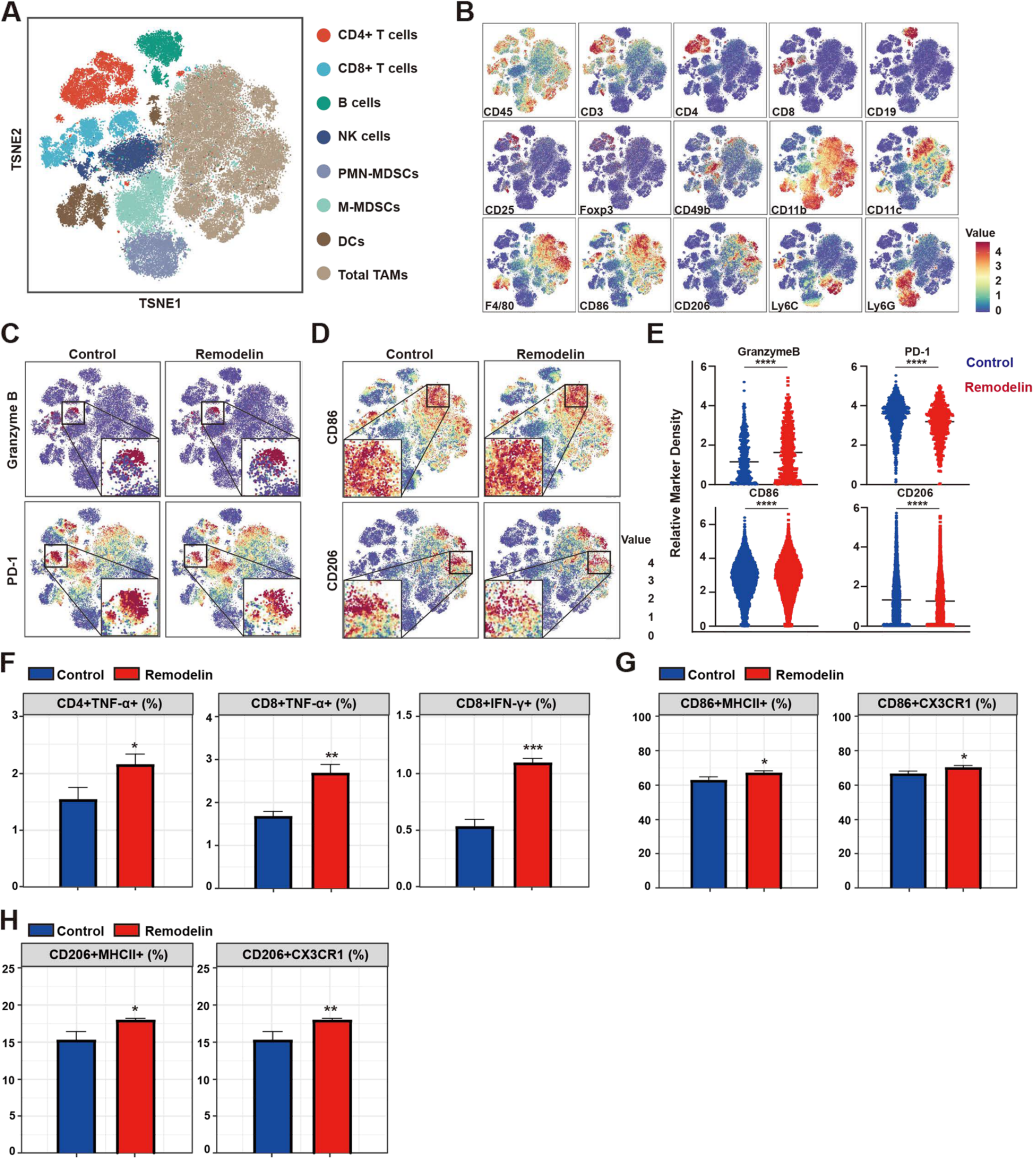
NAT10 function is required for immunosuppressive TME in TNBC. **A** t-SNE analysis of immune cells in 4T1 tumor, showing the identification of 8 main clusters. Each dot corresponds to a single cell, colored according to the cell clusters. **B** t-SNE subplot showing individual markers’ expression and distribution. **C**-**E** Normalized expression of Granzyme B, PD-1, CD86, and CD206 is shown by the t-SNE plot. **F**–**H** Quantification of subgroup changes in T cell and macrophage populations. The data are shown as the means ± SDs; *P < 0.05, **P < 0.01, ***P < 0.001, ****P < 0.0001

To elucidate the impact of NAT10 on tumor immunity, CyTOF analysis was performed on 4T1 tumor tissues. t-Distributed stochastic neighbor embedding (t-SNE) analysis of CyTOF data revealed 8 distinct intertumoral immune cell populations, with the majority comprising T cells and macrophages (Fig. 3A, B and Supplementary Fig. S4D). Of note, differences were noted in the activation state of immune infiltrates between tumors harvested from mice treated with remodelin and those sampled from the control group. Specifically, the expression level of the activation markers Granzyme and CD86 was higher in T cells and macrophages, respectively. On the other hand, the expression of the exhaustion marker PD-1 and that of CD206 was lower in T cells and macrophages, respectively (Fig. 3C-E). Specifically, the proportion of activated T cells, including CD4^+^TNF-α^+^, CD8^+^TNF-α^+^, and CD8^+^IFN-γ^+^, was significantly increased (Fig. 3F). Following this, macrophage activation markers were compared, uncovering minimal difference in the proportion of MHC II^+^ and CX3CR1^+^ cells between the remodelin and control groups (Fig. 3G, H). Importantly, the cytokine expression data (Supplementary Fig. S5A, B) suggested a more inflamed TME. The results of serum cytokine panels revealed significantly increased levels in various T cell activation cytokines (IL-12, IL-17, IFN-γ, and TNF-α), and decreased levels of cytokines (IL-10, IL-4, and IL-13) inhibiting T cell activation. These results were consistent with the mRNA expression profiles of tumor tissues (Supplementary Fig. S5C,  D). Overall, these results indicated that the NAT10 contributes to an immunosuppressive TME by inhibiting immune cell infiltration and activation, especially T cells.

### The loss of NAT10 facilitates T-cell infiltration and activation.

**Fig. 4:**
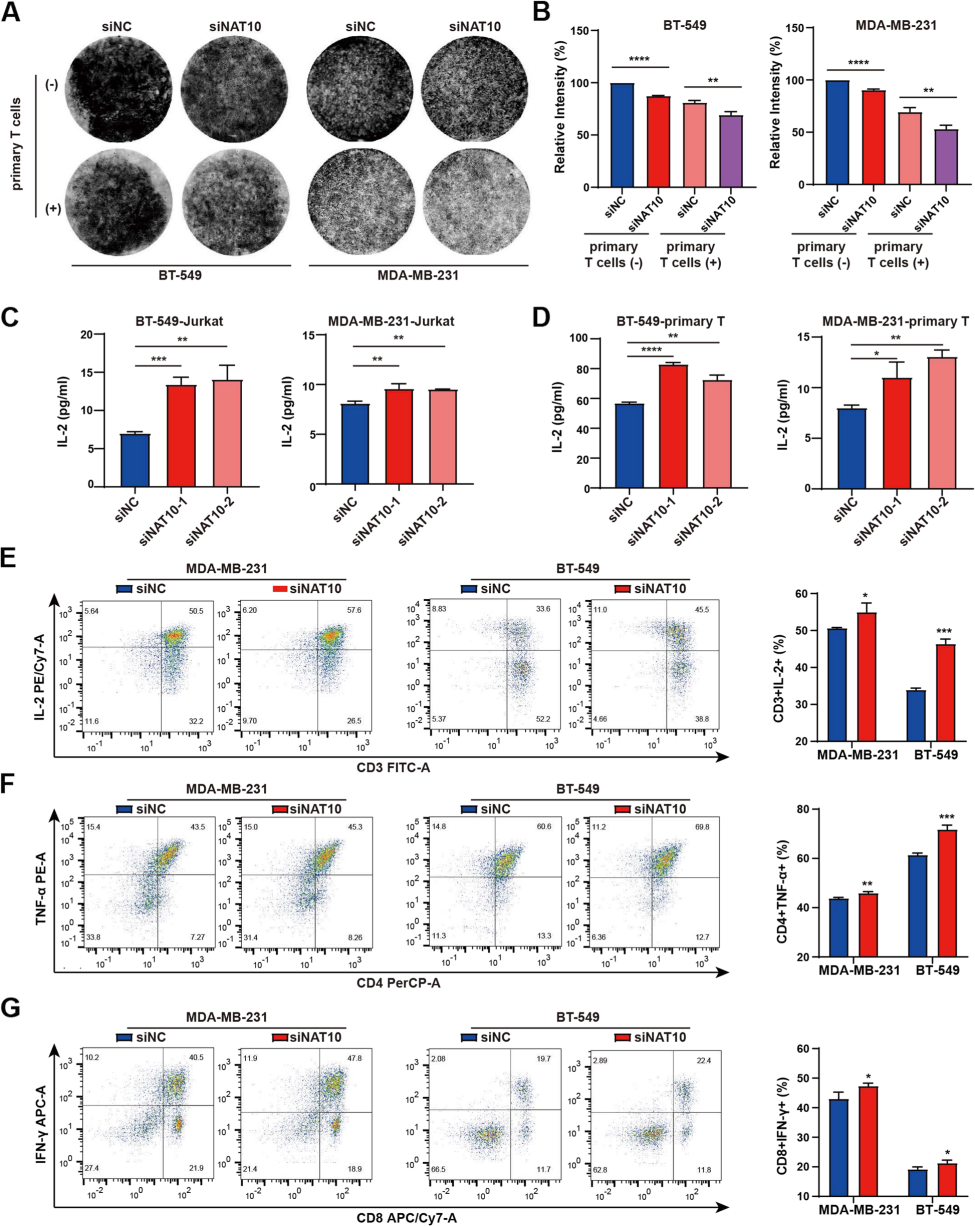
The loss of NAT10 facilitates T cell infiltration and activation. **A**, **B** T-cell cytotoxicity test by colony formation assay, and survival relative to the control is shown. **C**, **D** CM from NAT10-Kd cells were used to culture Jurkat and primary T cells, and ELISA detected IL-2 secretion. **E**–**G** Intracellular cytokine staining of IL-2, TNFα, and IFN-γ of T cell after co-culture with CM from NAT10-Kd cells. The data are shown as the means ± SDs; *P < 0.05, **P < 0.01, ***P < 0.001, ****P < 0.0001

To determine whether NAT10 inhibits T cell’ function, we initially employed in vitro co-culture assays. NAT10-Kd cells were more vulnerable to T cell-mediated killing than control.

cells (Fig. 4A, B). Afterward, the degree of T-cell activation was determined based on the level of IL-2 in the conditional medium (CM) of the NAT10-Kd and control cells. The results showed that T cells cultured in the CM derived from NAT10-Kd cells generated higher levels of IL-2 (Fig. 4C, D). In addition, the proportion of CD4^+^ and CD8^+^ T cells, detected by flow cytometry, was marginally higher in the NAT10-kd group (Supplementary Fig. S6A, B). Specifically, in the NAT10-kd group CM, T cells exhibited strong production of IL-2, TNF-α, and IFN-γ (Fig. 4E-G), in line with the results of CyTOF (Fig. 3F). Taken together, these results suggested that tumor-intrinsic NAT10 functions as a suppressive molecule that restricts T cell activation and effector states. However, the loss of NAT10 did not affect promoting macrophage activation (Supplementary Fig. S7).

### NAT10 contributes to the upregulation of ac4C modification in JunB mRNA

**Fig. 5:**
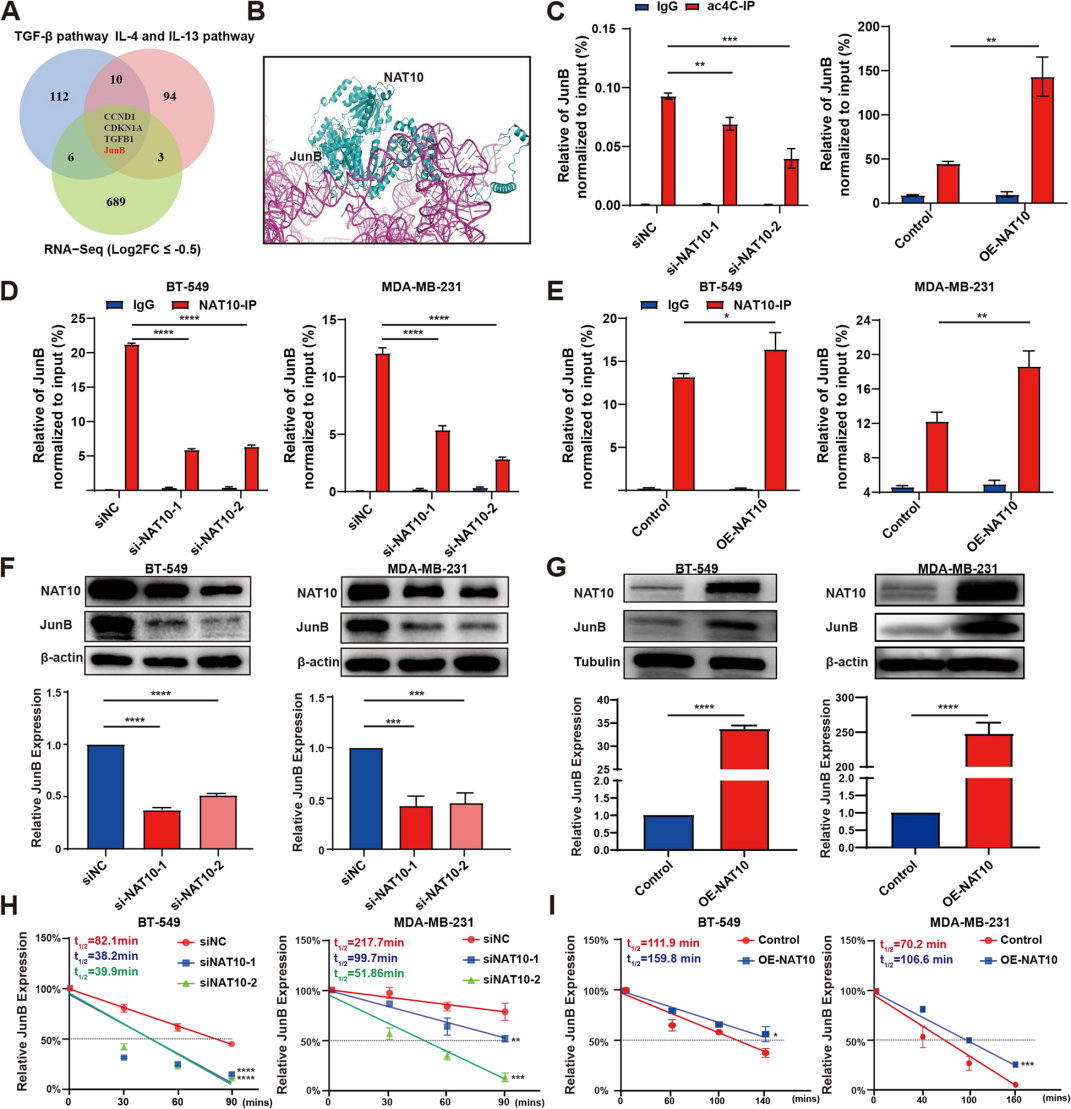
NAT10 contributes to the upregulation of ac4C modification in JunB mRNA. **A** Venn diagram showed the integration results of RNA-seq, IL-4, and IL-13 signaling pathway and TGF-β signaling pathway. **B** Molecular docking showing the interaction between NAT10 and JunB. **C** The relative levels of ac4C in JunB were tested by acRIP-qPCR in TNBC cells with NAT10 knockdown or overexpression. **D**, **E** The interaction between NAT10 and JunB mRNA was analyzed by RIP-qPCR assay in TNBC cells with NAT10 knockdown or overexpression. **F**, **G** mRNA and protein level of JunB upon NAT10 knockdown or overexpression. **H**, **I** RNA decay experiment validated JunB mRNA stability upon NAT10 knockdown or overexpression. The data are shown as the means ± SDs; *P < 0.05, **P < 0.01, ***P < 0.001, ****P < 0.0001

Furthermore, RNA-sequencing analysis was conducted to reveal the molecular mechanisms by which loss of NAT10 promotes T cell activation within TNBC cells. Integration of the results of RNA-seq (the down-regulated gene) with two gene sets (T cell inhibition-related signaling pathways) led to the identification of 4 genes (Fig. 5A). At the same time, the results of molecular docking analysis indicated a potential interaction between NAT10 protein and JunB mRNA (Fig. 5B). Subsequently, the PACES website was employed to predict targeted genes containing ac4C modification on mRNA [36], which illustrated that JunB had an ac4C modification at CAACAGCAACGGCGT (Supplementary Fig. S8A). Therefore, we speculated that NAT10 regulated the expression of JunB via ac4C modification. acRIP followed by qPCR confirmed the abundance of ac4C modifications on JunB mRNA (Fig. 5C). Further, the interaction between NAT10 and JunB was altered following NAT10 knockdown and overexpression, contributing to stronger binding between NAT10 and JunB (Fig. 5D, E). JunB expression was detected via qRT-PCR and WB following NAT10 knockdown and overexpression (Fig. 5F, G). In addition, RNA decay assays were performed to assess the effects of NAT10-regulated mRNA ac4C modification on JunB mRNA expression. The results showed that NAT10 deficiency decreased JunB mRNA stability (Fig. 5H, I). Puromycin uptake and RNA pulldown assay confirmed NAT10 deficiency inhibited translation rates and protein synthesis (Supplementary Fig. S8B), in agreement with the finding of a previous study that described that ac4C modification on mRNA upregulates the expression level of the targeted gene [25]. Collectively, these results indicated that NAT10 regulated JunB mRNA via ac4C modification.

### The loss of JunB contributes to LDHA downregulation

**Fig. 6:**
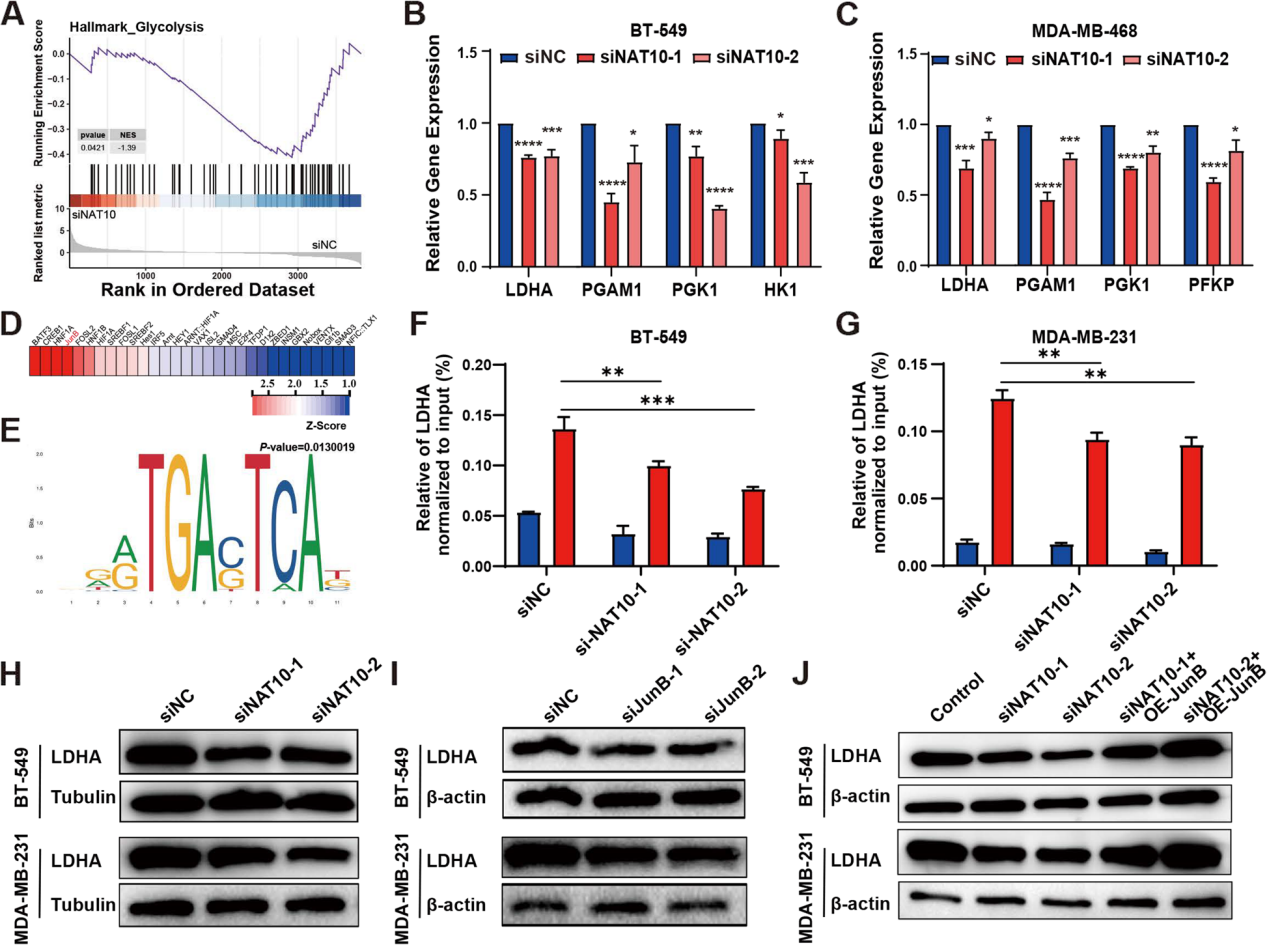
The loss of JunB contributes to the LDHA downregulation. **A** GSEA to assess specific enrichment of glycolysis signaling pathway upon RNA-seq (siNAT10 VS siNC). NES, normalized enrichment score; p-value was calculated with a permutation test. **B**, **C** qRT-PCR showed the mRNA expression of glycolysis-related gene after NAT10 knockdown. **D** Identification of transcription factors interacting with LDHA promoter by Pscan (http://159.149.160.88/pscan/) online promoter database. **E** The motif of JunB binding LDHA promoter, prompted by Pscan. **F**, **G** ChIP–qPCR assay to detect the enrichment of JunB at the promoter of the LDHA locus. **H**-**J** LDHA expression upon the NAT10 and JunB knockdown, and JunB overexpression. The data are shown as the means ± SDs; *P < 0.05, **P < 0.01, ***P < 0.001, ****P < 0.0001

Based on the RNA-seq results, Gene Set Enrichment Analysis (GSEA) showed that the glycolysis signaling pathway had a higher normalized enrichment score (NES) (Fig. 6A). Meanwhile, JunB has been reported to regulate glycolysis-related genes [37]. Thus, qRT-PCR was conducted to evaluate the effect of down-regulating the expression of NAT10 on the expression of multiple glycolysis-related genes (Fig. 6B, C). Given that the enhancement of tumor glycolysis can inhibit the activation of various immune cells in the immune microenvironment, we hypothesized that targeting NAT10-JunB could inhibit the secretion of lactate by TNBC cells, thereby promoting T activation. The Pscan website was used to identify glycolysis-related genes regulated by the transcription factor JunB, and the results suggested that JunB may bind to the LDHA promoter (Fig. 6D, E). Next, ChIP experiments were performed, and the results corroborated that JunB may bind to the LDHA promoter (Fig. 6F, G). Our results also confirmed that LDHA expression was significantly downregulated following NAT10 (Fig. 6H) and JunB knockdown (Fig. 6I and Supplementary Fig. S8C, D). In addition, JunB overexpression (JunB-OE) reversed the down-regulation in LDHA expression after NAT10 knockdown (Fig. 6J). Similarly, treating TNBC cells with different concentrations of remodelin also resulted in significant downregulation of LDHA expression (Supplementary Fig. S8E). These results revealed a novel mechanism by which NAT10-JunB-LDHA may contribute to the elevation of glycolysis in TNBC.

### NAT10 contributed to glycolysis elevation and T-cell inhibition by upregulating JunB expression

**Fig. 7:**
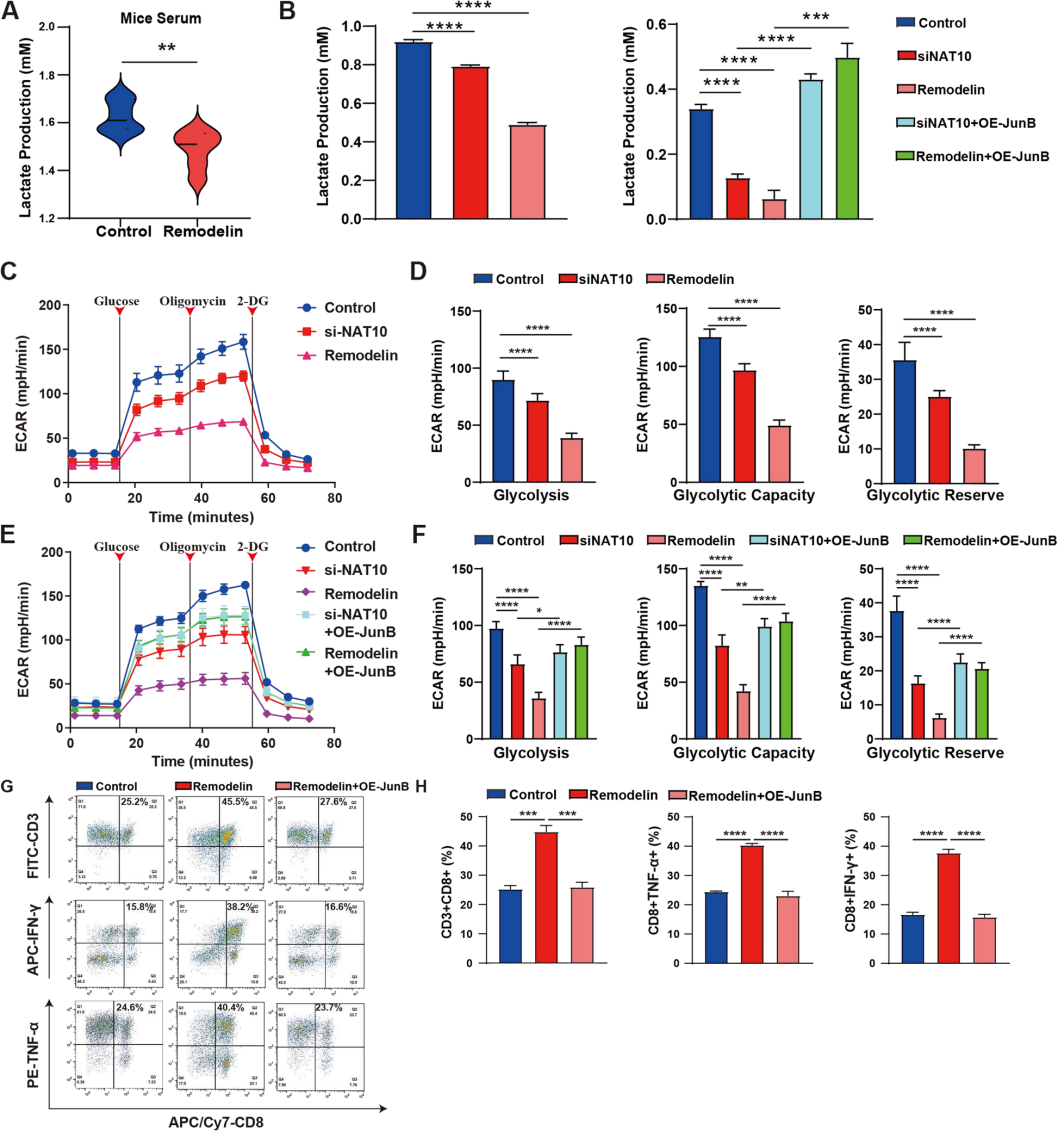
NAT10 contributed to glycolysis elevation and T-cell inhibition by upregulating JunB expression. **A** Quantification of lactate production in control and remodelin treatment of mice serum. **B** Quantification of lactate production in the supernatant. **C**-**F** Glycolytic stress tests using the Seahorse XF bioanalyzer to measure the glycolysis level, glycolytic capacity, and reserve. **G**, **H** Primary T cell was incubated with CM from NAT10-Kd or JunB-OE cells. T-cell activation was detected by flow cytometry. The data are shown as the means ± SDs; *P < 0.05, **P < 0.01, ***P < 0.001, ****P < 0.0001

It is well established that tumors utilize glycolysis to promote growth, glucose consumption, lactate secretion, and restrict T cell activation, thereby promoting tumor progression and immune evasion [38, 39]. Thus, to determine whether NAT10 inhibits T-cell activation by promoting glycolysis, lactate concentrations in the serum of mice were measured, and the results demonstrated that the level of lactate was lower in the remodelin treatment group (Fig. 7A). The loss of NAT10 inhibited the production of lactate in vitro, whilst JunB-OE partially restored lactate levels in NAT10-Kd cells (Fig. 7B). To examine the effect of NAT10 and JunB on glycolytic metabolism, glycolysis levels were determined using a seahorse instrument, which showed decreased glycolytic levels, capacity, and reserve in NAT10-Kd cells compared with control cells (Fig. 7C, D). Moreover, overexpressing JunB reversed the downregulation of glycolysis (Fig. 7E, F). Meanwhile, the loss of NAT10 decreased ATP levels and the NAD + /NADH ratio (Supplementary Fig. S9A-C). Thus, human primary T cells were co-incubated in the CM collected from NAT10-Kd or JunB-OE cells. Strikingly, the levels of cytokines such as TNF-α and IFN-γ, as well as the number of CD8 + T cells, were lower in JunB-OE cells (Fig. 7G-H). These results demonstrated that NAT10 promoted glycolysis in TNBC cells and suppressed T-cell activation by regulating the NAT10-JunB-LDHA signaling pathway.

### *Remodelin potentiates anti-CTLA-4 efficacy *in vivo*.*

**Fig. 8:**
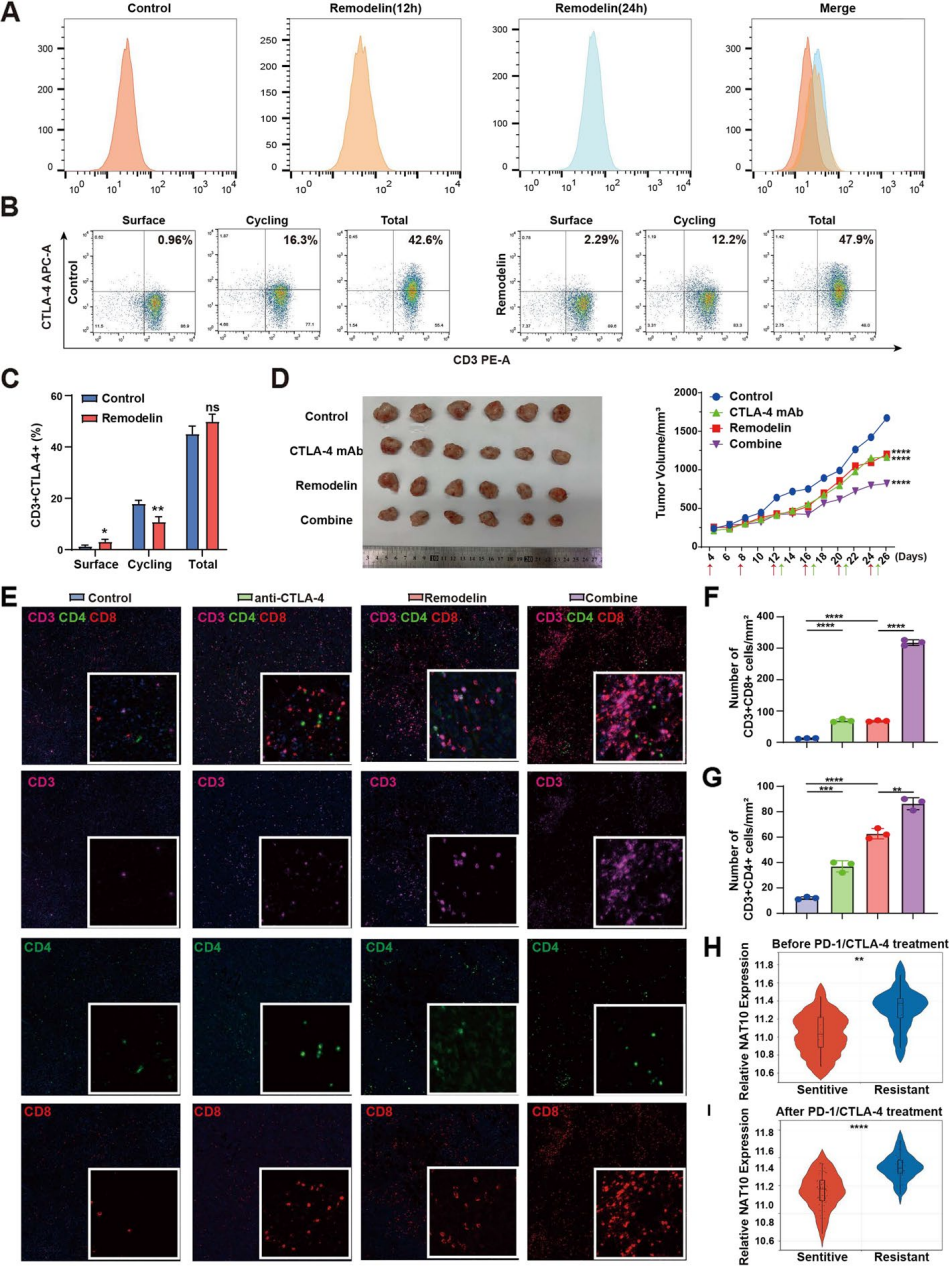
Remodelin potentiates anti-CTLA-4 efficacy in vivo. **A** Representative changes in membrane CTLA-4 expression, evaluated by flow cytometry analysis on primary T cell. **B**, **C** The constitutive cycling of CTLA-4 was measured by flow cytometry. CTLA-4 was stained at 4 °C (Surface), at 37 °C for 2 h (Cycling), or on fixed and permeabilized cells (Total). **D** Tumor volumes were determined on the indicated days with different treatments in 4T1 mice (*n* = 6). **E**–**G** The markers on T cell were detected by mIHC in mice tumor tissues. Data are presented as means from three independent experiments ± S.D. **H**, **I** NAT10 expression of the sensitive and resistant group before or after PD-1/CTLA-4 treatment. The data are shown as the means ± SDs; *P < 0.05, **P < 0.01, ***P < 0.001, ****P < 0.0001, ns, nonsignificant

Roberta Z. et al. demonstrated that anti-CTLA-4 yielded superior therapeutic outcomes in mice bearing glycolysis-defective tumors [40]. Consequently, the synergistic effect of the combination of remodelin with anti-CTLA-4 therapy was investigated in TNBC. Further analysis of CyTOF unveiled that the expression level of CTLA-4 on T cells was increased in the remodelin treatment group (Supplementary Fig. S10A). At the same time, the expression level of CTLA-4 on the surface of regulatory T cells (Tregs) was increased, whilst the number of Treg cells remained unaltered (Supplementary Fig. S10B, C). Similarly, the expression of CTLA-4 significantly increased on the surface of T cells after remodelin treatment (Fig. 8A). Thereafter, the dynamics of surface, cycling, and total CTLA-4 were evaluated in human primary T cells. The results exposed that the expression level of CTLA-4 was increased on the surface of CD3^+^T. With the total CTLA-4 pool unchanged, remodelin up-regulated the membrane expression of CTLA-4 while inhibiting circulating CTLA-4 (Fig. 8B, C). Besides, the expression level of CTLA-4 was lower in breast cancer cells compared with that in tumors, indicating a better therapeutic response to CTLA-4 mAb (Supplementary Fig. S10D). This mechanism can potentially increase the therapeutic effect of CTLA-4 mAb on breast cancer.

Subsequently, the efficacy of remodelin combined with anti-CTLA-4 mAb was assessed in vivo. The combination demonstrated significantly higher inhibition of tumor growth compared to remodelin alone or CTLA-4 blockade alone (Fig. 8D). Multicolor fluorescence IHC assays further confirmed a significant increase in the number of CD4^+^ and CD8^+^ T cells after combination treatment (Fig. 8E-G). Evaluating the relationship between ICBs resistance and NAT10 expression through the GSEA database revealed that NAT10 was highly expressed in immunotherapy (PD-1/CTLA-4) resistant models compared to immunotherapy-sensitive models [41] (Fig. 8H, I). These results indicated that NAT10 is associated with tumor immune suppression microenvironment traits and ICB resistance. Collectively, these results demonstrated that remodelin treatment increased the functionality and number of effector T cells, with these effects being more pronounced using the combination of anti-CTLA-4 mAb and remodelin.

## Discussion

Epigenetic alterations play a vital role in the progression of various tumors [42]. The ac4C modification of mRNA recently garnered extensive attention in the epigenetic field [43–45]. Preliminary results showed that RNA ac4C modification affects tumorigenesis, prognosis, and immune cell infiltration in patients with TNBC [46]. Currently, numerous studies have focused on the regulation of immune checkpoint expression by oncogenes, thereby affecting the effect of immunotherapy [47, 48]. Distinct from previous studies, our study demonstrated that the loss of NAT10 elicited anti-tumorigenic effects through mechanisms that are both tumor cell-intrinsic (inhibiting TNBC glycolysis level and progression) and -extrinsic (increasing T cell infiltration and activation), transforming an immunosuppressive “cold tumor” to an immune-activated “hot tumor”.

The results of RNA-seq analysis, immunosuppressive gene sets, and ac4C modification site prediction suggested that NAT10 up-regulated JunB expression by enhancing ac4C modification on JunB mRNA. It is reported that NAT10 can promote the glycolysis level in the gastric and cervical cancer [43, 45]. Meanwhile, MYC family and bZIP family are known to transcriptionally activate genes for glycolysis enzymes [49]. c-Jun, C/EBPb, and JunB, which belong to the bZIP family, has been proven to be able to promote glycolysis enzymes expression [37]. As tumors exhibit high glycolytic activity, abundant secretion of lactate from tumor cells creates an acidic immune microenvironment that inhibits immune cell cytotoxicity and effector functions [50, 51]. Our findings validated that the loss of NAT10 promoted T cell activation, which was reversed by JunB-OE in TNBC (Fig. 7G-H). These findings highlighted the critical regulatory role of mRNA ac4C modification in driving JunB-mediated glycolysis addiction and suppressive TME, identifying NAT10 as a potential therapeutic target for cancer immunotherapy in TNBC. More importantly, the loss of NAT10 enhanced the sensitivity to anti-CTLA-4 therapy by impeding the constitutive cycling of CTLA-4 and increasing the surface expression of T cell. Qin G etc. also demonstrated that NAT10 knockdown can inhibit PD-L1 expression and improves the response to PD-L1 mAb [47]. These results showed that NAT10 is expected to be a target for cancer immunotherapy.

CTLA-4 acts as a suppressive molecule that inhibits both the proliferation and the function of T cells [52]*.* It inhibits T cell activation by outcompeting CD28 ligand binding and recruiting phosphatases in its cytoplasmic tail. Upregulation of CTLA-4 and PD-1 is generally observed during T-cell activation, preventing damage from an excessive immune response. Therefore, we speculate that remodelin promotes the up-regulation of CTLA-4 expression on T cells, owing to its role in promoting T cell activation. Multiple studies have established that CTLA-4 blockade can be exploited to treat glycolysis-low tumors or in combination with inhibitors of tumor glycolysis [47, 53, 54]. To date, Ipilimumab, a CTLA-4 mAb, has proven efficacy in the treatment of certain tumors, such as melanoma [55], non-small cell lung cancer [56], and renal cell carcinoma [57]. However, ipilimumab requires a longer treatment duration to achieve an effective antitumor response, compared to other currently used agents. This delayed onset of action allows for disease progression, which could result in more adverse events [58]. Breast cancer has traditionally been considered a poorly immunogenic “cold tumor”, which leads to a poor response to CTLA-4 mAb in breast cancer patients [59]. To date, The results of multiple clinical trials in breast cancer, with tremelimumab and ipilimumab, have shown negative results both in terms of progression-free survival and overall survival [60]. Therefore, remodelin treatment can activate the TME and address the delayed response of CTLA-4 mAb before considering combination therapy with CTLA-4 mAb.

Nevertheless, some limitations of this study merit acknowledgment. To begin, remodeling-treatment promoted macrophage activation in mouse tumor tissue. However, no significant activation of macrophage co-cultured with the CM from NAT10-kd TNBC cells was noted (Supplementary Fig. S7B). We postulate that NAT10 may affect the levels of other cytokines or factors secretion and influence macrophage activation. NAT10 may play a role in maintaining the immune suppressive phenotype in macrophages. Secondly, the reason why remodelin did not affect the glycolysis level of T cells and macrophages, except for M1-like macrophages were not investigated (Supplementary Fig. S9D, E). Thus, the role of NAT10 in T cells and macrophages warrants further investigation.

## Conclusions

In summary, the loss of NAT10 preserved suppressive TME of TNBC by limiting glycolysis and promoting T cell infiltration and activation. Further, the combination of remodelin and CTLA-4 blockade improves the limited therapeutic efficacy of monotherapies to elicit potent antitumor immunity, necessitating further research and clinical trials. Altogether, our study uncovered that RNA could operate as an additional layer of genetic regulation for immune evasion, thereby laying a theoretical reference for the discovery of a new class of potentially vulnerable targets for immunotherapy.

## Supplementary information

Supplementary Material 1: Supplementary Data 1. Reagent information

Supplementary Material 2: Supplementary Data 2. Information from HMUCC patients' tissues

Supplementary Material 3: Supplementary Data 3. Information from TCGA patients' tissues

Supplementary Material 4: Supplementary Data 4. Data from GSE124821

Supplementary Material 5: Supplementary Data 5. CyTOF antibody information

Supplementary Material 6: Supplementary Data 6. Sequence of primers and siRNA of NAT10

Supplementary Material 7: Supplementary Fig. S1. Ac4C level in TNBC tissues and efficiency of targeting NAT10. (A) Heat map showed the expression of NAT10 in different molecular types. (B)The ac4C level in TNBC tissues was determined by dot blot. (C) The NAT10 expression in different breast cancer cell lines. (D-F) Western blot and qRT-PCR measured NAT10 expression by using siRNA and remodelin. (G) Dot-blot quantification of ac4C abundance in mRNA transcripts. Ac4C dot blot assay was performed with methylene blue (MB) as a loading control. The data are shown as the means ± SDs; ****P < 0.0001. Supplementary Fig. S2. NAT10 facilitates the TNBC tumorigenesis.  (A-D) NAT10 overexpression promotes the TNBC cells invasion and migration. (E) NAT10 overexpression promotes the TNBC cells proliferation. The data are shown as the means ± SDs; ****P < 0.0001. Supplementary Fig. S3. NAT10 facilitates the 4T1 cell development. (A) The loss of NAT10 inhibits the 4T1 proliferation. (B-E) The loss of NAT10 inhibits the 4T1 invasion and migration. The data are shown as the means ± SDs; ***P < 0.001, ****P < 0.0001. Supplementary Fig. S4. NAT10 function is required for immunosuppressive TME in TNBC. (A) NAT10 expression in BLIS and non-immunosuppressive subtype (Others), generated from TCGA database (B) Correlation of NAT10 expression with immune infiltration levels in breast cancer based on the TIMER2 analysis. (C) Kaplan-Meier survival curves comparing immune cell infiltration of breast cancer in TIMER2. (D) Heatmap of phenograph clusters of CD45+ cells. The relative expression levels of markers across cells were shown and sorted by cell type. The data are shown as the means ± SDs; *P < 0.05. Supplementary Fig. S5. Targeting NAT10 increases cytokines that could activate T cell and macrophages. (A-B) Heatmap showing serum cytokine concentration of remodeling-treated mice bearing 4T1 tumor compared with control. (C-D) Mice tissues were used to detect the mRNA expression of cytokines. The data are shown as the means ± SDs; *P< 0.05, **P < 0.01, ***P < 0.001, ****P < 0.0001, ns, nonsignificant. Supplementary Fig. S6. The loss of NAT10 facilitates T cell infiltration.. (A-B) Quantification of CD4^+^ and CD8^+^ populations among CD3^+^ T cells after co-culture with remodelin-treated TNBC cells. The data are shown as the means ± SDs; *P< 0.05, **P < 0.01. Supplementary Fig. S7. The loss of NAT10 does not affect the macrophage activation. (A) THP-1 was induced and differentiated into M1- and M2-like macrophages. (B) CM from NAT10-Kd cells were used to culture M1- and M2-like macrophages. Supplementary Fig. S8. NAT10 contributed to LDHA upregulating by interacting with JunB mRNA. (A)The sequence on JunB mRNA containing ac4C modification. (B) RNA-pulldown detected the protein interacted with JunB mRNA. Cells were treated with 1mM puromycin at 37℃ for 30 min, followed by protein isolation and western blotting. (C-D) qRT-PCR and western blot measured expression efficiency upon the knockdown and overexpression of JunB. (E) LDHA expression upon remodelin treatment. The data are shown as the means ± SDs; **P< 0.01, ****P < 0.0001. Supplementary Fig. S9. NAT10 did not affect the expression of LDHA and lactate production in T cells and macrophages. (A) Quantification of ATP production in control and NAT10-Kd cells. (B-C) Quantification of NAD+ and NADH in control and NAT10-Kd cells. (D) Jurkat was treated with remodelin, and LDHA expression and lactate production were measured. (E) M1- and M2-like macrophages were treated with remodelin, and LDHA expression and lactate production were measured. The data are shown as the means ± SDs; **P < 0.01, ***P < 0.001,****P < 0.0001, ns, nonsignificant. Supplementary Fig. S10. Remodelin promotes the expression of CTLA-4 on T cells. (A) CTLA-4 expression on CD8^+^ and CD4^+^ T cells was measured based on the CyTOF results. (B-C) The number of Treg and CTLA-4^+^ Treg were measured based on the CyTOF results. (D) The expression of CLTA-4 in four tumor tissues is based on the TCGA database. The data are shown as the means ± SDs; *P < 0.05, ****P < 0.0001, ns, nonsignificant. Supplementary Fig. S11. Molecular mechanism

## Data Availability

The data that support the findings of this study are available from the corresponding author upon reasonable request. The sequencing data are available in Gene Expression Omnibus under accession number GSE124821 and GSE96058.

## Abbreviations

ac4C: N4-Acetylcytidine
TNBC: Triple negative breast cancer
TME: Tumor microenvironment
PD-1: Programmed cell death protein 1
PD-L1: Programmed cell death 1 ligand 1
ICBs: Immune checkpoint inhibitors
CTLA-4: Cytotoxic T lymphocyte associate protein-4
IL-10: Interleukin-10
CyTOF: Mass cytometry by time of flight
HMUCC: Harbin Medical University Cancer Center
LPS: Lipopolysaccharide
acRIP: Ac4C immunoprecipitation
ChIP: Chromatin-immunoprecipitation
ELISA: Enzyme-linked immunosorbent assay
IHC: Immunohistochemistry analysis
TIMER2: Tumor Immune Estimation Resource 2
GESA: Gene Set Enrichment Analysis

## References

[1] Siegel RL, Miller KD, Fuchs HE, Jemal A. Cancer Statistics, 2021. CA Cancer J Clin. 2021;71(1):7–33. 33433946 10.3322/caac.21654

[2] Sharma P. Update on the Treatment of Early-Stage Triple-Negative Breast Cancer. Curr Treat Options Oncol. 2018;19(5):22. 29656345 10.1007/s11864-018-0539-8

[3] Khan MA, Jain VK, Rizwanullah M, Ahmad J, Jain K. PI3K/AKT/mTOR pathway inhibitors in triple-negative breast cancer: a review on drug discovery and future challenges. Drug Discov Today. 2019;24(11):2181–91. 31520748 10.1016/j.drudis.2019.09.001

[4] Jyotsana N, Zhang Z, Himmel LE, Yu F, King MR. Minimal dosing of leukocyte targeting TRAIL decreases triple-negative breast cancer metastasis following tumor resection. Sci Adv. 2019;5(7):eaaw4197. 31355333 10.1126/sciadv.aaw4197 PMC6656540

[5] Emens LA. Immunotherapy in Triple-Negative Breast Cancer. Cancer J. 2021;27(1):59–66. 33475294 10.1097/PPO.0000000000000497

[6] Adams S, Loi S, Toppmeyer D, Cescon DW, De Laurentiis M, Nanda R, et al. Pembrolizumab monotherapy for previously untreated, PD-L1-positive, metastatic triple-negative breast cancer: cohort B of the phase II KEYNOTE-086 study. Ann Oncol. 2019;30(3):405–11. 30475947 10.1093/annonc/mdy518

[7] Schmid P, Adams S, Rugo HS, Schneeweiss A, Barrios CH, Iwata H, et al. Atezolizumab and Nab-Paclitaxel in Advanced Triple-Negative Breast Cancer. N Engl J Med. 2018;379(22):2108–21. 30345906 10.1056/NEJMoa1809615

[8] Hodi FS, O’Day SJ, McDermott DF, Weber RW, Sosman JA, Haanen JB, et al. Improved survival with ipilimumab in patients with metastatic melanoma. N Engl J Med. 2010;363(8):711–23. 20525992 10.1056/NEJMoa1003466 PMC3549297

[9] Robert C, Thomas L, Bondarenko I, O’Day S, Weber J, Garbe C, et al. Ipilimumab plus dacarbazine for previously untreated metastatic melanoma. N Engl J Med. 2011;364(26):2517–26. 21639810 10.1056/NEJMoa1104621

[10] Braun DA, Bakouny Z, Hirsch L, Flippot R, Van Allen EM, Wu CJ, et al. Beyond conventional immune-checkpoint inhibition - novel immunotherapies for renal cell carcinoma. Nat Rev Clin Oncol. 2021;18(4):199–214. 33437048 10.1038/s41571-020-00455-z PMC8317018

[11] Perets R, Bar J, Rasco DW, Ahn MJ, Yoh K, Kim DW, et al. Safety and efficacy of quavonlimab, a novel anti-CTLA-4 antibody (MK-1308), in combination with pembrolizumab in first-line advanced non-small-cell lung cancer. Ann Oncol. 2021;32(3):395–403. 33276076 10.1016/j.annonc.2020.11.020

[12] Peters S, Scherpereel A, Cornelissen R, Oulkhouir Y, Greillier L, Kaplan MA, et al. First-line nivolumab plus ipilimumab versus chemotherapy in patients with unresectable malignant pleural mesothelioma: 3-year outcomes from CheckMate 743. Ann Oncol. 2022;33(5):488–99. 35124183 10.1016/j.annonc.2022.01.074

[13] Topalian SL, Hodi FS, Brahmer JR, Gettinger SN, Smith DC, McDermott DF, et al. Safety, activity, and immune correlates of anti-PD-1 antibody in cancer. N Engl J Med. 2012;366(26):2443–54. 22658127 10.1056/NEJMoa1200690 PMC3544539

[14] De La Cruz LM, Czerniecki BJ. Immunotherapy for Breast Cancer is Finally at the Doorstep: Immunotherapy in Breast Cancer. Ann Surg Oncol. 2018;25(10):2852–7. 30014455 10.1245/s10434-018-6620-5

[15] Solinas C, Gombos A, Latifyan S, Piccart-Gebhart M, Kok M, Buisseret L. Targeting immune checkpoints in breast cancer: an update of early results. ESMO Open. 2017;2(5): e000255. 29177095 10.1136/esmoopen-2017-000255 PMC5687552

[16] Ledys F, Kalfeist L, Galland L, Limagne E, Ladoire S. Therapeutic Associations Comprising Anti-PD-1/PD-L1 in Breast Cancer: Clinical Challenges and Perspectives. Cancers (Basel). 2021;13(23):5999. 10.3390/cancers13235999 PMC8656936 34885109

[17] Segovia-Mendoza M, Morales-Montor J. Immune Tumor Microenvironment in Breast Cancer and the Participation of Estrogen and Its Receptors in Cancer Physiopathology. Front Immunol. 2019;10:348. 30881360 10.3389/fimmu.2019.00348 PMC6407672

[18] Brand A, Singer K, Koehl GE, Kolitzus M, Schoenhammer G, Thiel A, et al. LDHA-Associated Lactic Acid Production Blunts Tumor Immunosurveillance by T and NK Cells. Cell Metab. 2016;24(5):657–71. 27641098 10.1016/j.cmet.2016.08.011

[19] Ouyang W, Rutz S, Crellin NK, Valdez PA, Hymowitz SG. Regulation and functions of the IL-10 family of cytokines in inflammation and disease. Annu Rev Immunol. 2011;29:71–109. 21166540 10.1146/annurev-immunol-031210-101312

[20] Travis MA, Sheppard D. TGF-beta activation and function in immunity. Annu Rev Immunol. 2014;32:51–82. 24313777 10.1146/annurev-immunol-032713-120257 PMC4010192

[21] Chang CH, Pearce EL. Emerging concepts of T cell metabolism as a target of immunotherapy. Nat Immunol. 2016;17(4):364–8. 27002844 10.1038/ni.3415 PMC4990080

[22] Shen Q, Zheng X, McNutt MA, Guang L, Sun Y, Wang J, et al. NAT10, a nucleolar protein, localizes to the midbody and regulates cytokinesis and acetylation of microtubules. Exp Cell Res. 2009;315(10):1653–67. 19303003 10.1016/j.yexcr.2009.03.007

[23] Lv J, Liu H, Wang Q, Tang Z, Hou L, Zhang B. Molecular cloning of a novel human gene encoding histone acetyltransferase-like protein involved in transcriptional activation of hTERT. Biochem Biophys Res Commun. 2003;311(2):506–13. 14592445 10.1016/j.bbrc.2003.09.235

[24] Jin G, Xu M, Zou M, Duan S. The Processing, Gene Regulation, Biological Functions, and Clinical Relevance of N4-Acetylcytidine on RNA: A Systematic Review. Mol Ther Nucleic Acids. 2020;20:13–24. 32171170 10.1016/j.omtn.2020.01.037 PMC7068197

[25] Arango D, Sturgill D, Alhusaini N, Dillman AA, Sweet TJ, Hanson G, et al. Acetylation of Cytidine in mRNA Promotes Translation Efficiency. Cell. 2018;175(7):1872-86 e24. 30449621 10.1016/j.cell.2018.10.030 PMC6295233

[26] Xie L, Zhong X, Cao W, Liu J, Zu X, Chen L. Mechanisms of NAT10 as ac4C writer in diseases. Mol Ther Nucleic Acids. 2023;32:359–68. 37128278 10.1016/j.omtn.2023.03.023 PMC10148080

[27] Cheng HP, Yang XH, Lan L, Xie LJ, Chen C, Liu C, et al. Chemical Deprenylation of N(6) -Isopentenyladenosine (i(6) A) RNA. Angew Chem Int Ed Engl. 2020;59(26):10645–50. 32198805 10.1002/anie.202003360

[28] Feng Z, Li K, Qin K, Liang J, Shi M, Ma Y, et al. The LINC00623/NAT10 signaling axis promotes pancreatic cancer progression by remodeling ac4C modification of mRNA. J Hematol Oncol. 2022;15(1):112. 35978332 10.1186/s13045-022-01338-9 PMC9387035

[29] Zhang Y, Jing Y, Wang Y, Tang J, Zhu X, Jin WL, et al. NAT10 promotes gastric cancer metastasis via N4-acetylated COL5A1. Signal Transduct Target Ther. 2021;6(1):173. 33941767 10.1038/s41392-021-00489-4 PMC8093205

[30] Qi P, Chen YK, Cui RL, Heng RJ, Xu S, He XY, et al. Overexpression of NAT10 induced platinum drugs resistance in breast cancer cell. Zhonghua Zhong Liu Za Zhi. 2022;44(6):540–9. 35754228 10.3760/cma.j.cn112152-20211231-00986

[31] Liu HY, Liu YY, Yang F, Zhang L, Zhang FL, Hu X, et al. Acetylation of MORC2 by NAT10 regulates cell-cycle checkpoint control and resistance to DNA-damaging chemotherapy and radiotherapy in breast cancer. Nucleic Acids Res. 2020;48(7):3638–56. 32112098 10.1093/nar/gkaa130 PMC7144926

[32] Dalhat MH, Mohammed MRS, Ahmad A, Khan MI, Choudhry H. Remodelin, a N-acetyltransferase 10 (NAT10) inhibitor, alters mitochondrial lipid metabolism in cancer cells. J Cell Biochem. 2021;122(12):1936–45. 34605570 10.1002/jcb.30155

[33] Auwerx J. The human leukemia cell line, THP-1: a multifacetted model for the study of monocyte-macrophage differentiation. Experientia. 1991;47(1):22–31. 1999239 10.1007/BF02041244

[34] Daigneault M, Preston JA, Marriott HM, Whyte MK, Dockrell DH. The identification of markers of macrophage differentiation in PMA-stimulated THP-1 cells and monocyte-derived macrophages. PLoS ONE. 2010;5(1): e8668. 20084270 10.1371/journal.pone.0008668 PMC2800192

[35] Jiang YZ, Ma D, Suo C, Shi J, Xue M, Hu X, et al. Genomic and Transcriptomic Landscape of Triple-Negative Breast Cancers: Subtypes and Treatment Strategies. Cancer Cell. 2019;35(3):428-40 e5. 30853353 10.1016/j.ccell.2019.02.001

[36] Zhao W, Zhou Y, Cui Q, Zhou Y. PACES: prediction of N4-acetylcytidine (ac4C) modification sites in mRNA. Sci Rep. 2019;9(1):11112. 31366994 10.1038/s41598-019-47594-7 PMC6668381

[37] Liu Y, Liang G, Xu H, Dong W, Dong Z, Qiu Z, et al. Tumors exploit FTO-mediated regulation of glycolytic metabolism to evade immune surveillance. Cell Metab. 2021;33(6):1221-33 e11. 33910046 10.1016/j.cmet.2021.04.001

[38] Fischer K, Hoffmann P, Voelkl S, Meidenbauer N, Ammer J, Edinger M, et al. Inhibitory effect of tumor cell-derived lactic acid on human T cells. Blood. 2007;109(9):3812–9. 17255361 10.1182/blood-2006-07-035972

[39] Ohashi T, Akazawa T, Aoki M, Kuze B, Mizuta K, Ito Y, et al. Dichloroacetate improves immune dysfunction caused by tumor-secreted lactic acid and increases antitumor immunoreactivity. Int J Cancer. 2013;133(5):1107–18. 23420584 10.1002/ijc.28114

[40] Zappasodi R, Serganova I, Cohen IJ, Maeda M, Shindo M, Senbabaoglu Y, et al. CTLA-4 blockade drives loss of T(reg) stability in glycolysis-low tumours. Nature. 2021;591(7851):652–8. 33588426 10.1038/s41586-021-03326-4 PMC8057670

[41] Hollern DP, Xu N, Thennavan A, Glodowski C, Garcia-Recio S, Mott KR, et al. B Cells and T Follicular Helper Cells Mediate Response to Checkpoint Inhibitors in High Mutation Burden Mouse Models of Breast Cancer. Cell. 2019;179(5):1191-206 e21. 31730857 10.1016/j.cell.2019.10.028 PMC6911685

[42] Mazor T, Pankov A, Song JS, Costello JF. Intratumoral Heterogeneity of the Epigenome. Cancer Cell. 2016;29(4):440–51. 27070699 10.1016/j.ccell.2016.03.009 PMC4852161

[43] Chen X, Hao Y, Liu Y, Zhong S, You Y, Ao K, et al. NAT10/ac4C/FOXP1 Promotes Malignant Progression and Facilitates Immunosuppression by Reprogramming Glycolytic Metabolism in Cervical Cancer. Adv Sci (Weinh). 2023;10(32): e2302705. 37818745 10.1002/advs.202302705 PMC10646273

[44] Xu T, Wang J, Wu Y, Wu JY, Lu WC, Liu M, et al. Ac4C Enhances the Translation Efficiency of Vegfa mRNA and Mediates Central Sensitization in Spinal Dorsal Horn in Neuropathic Pain. Adv Sci (Weinh). 2023;10(35): e2303113. 37877615 10.1002/advs.202303113 PMC10724395

[45] Yang Q, Lei X, He J, Peng Y, Zhang Y, Ling R, et al. N4-Acetylcytidine Drives Glycolysis Addiction in Gastric Cancer via NAT10/SEPT9/HIF-1alpha Positive Feedback Loop. Adv Sci (Weinh). 2023;10(23): e2300898. 37328448 10.1002/advs.202300898 PMC10427357

[46] Zhang X, Zeng J, Wang J, Yang Z, Gao S, Liu H, et al. Revealing the Potential Markers of N(4)-Acetylcytidine through acRIP-seq in Triple-Negative Breast Cancer. Genes (Basel). 2022;13(12):2400. 10.3390/genes13122400 PMC9777589 36553667

[47] Qin G, Bai F, Hu H, Zhang J, Zhan W, Wu Z, et al. Targeting the NAT10/NPM1 axis abrogates PD-L1 expression and improves the response to immune checkpoint blockade therapy. Mol Med. 2024;30(1):13. 38243170 10.1186/s10020-024-00780-4 PMC10799409

[48] Wang Q, Li G, Ma X, Liu L, Liu J, Yin Y, et al. LncRNA TINCR impairs the efficacy of immunotherapy against breast cancer by recruiting DNMT1 and downregulating MiR-199a-5p via the STAT1-TINCR-USP20-PD-L1 axis. Cell Death Dis. 2023;14(2):76. 36725842 10.1038/s41419-023-05609-2 PMC9892521

[49] Wang Z, Huang Y, Lu W, Liu J, Li X, Zhu S, et al. c-myc-mediated upregulation of NAT10 facilitates tumor development via cell cycle regulation in non-small cell lung cancer. Med Oncol. 2022;39(10):140. 35834140 10.1007/s12032-022-01736-6

[50] Mendler AN, Hu B, Prinz PU, Kreutz M, Gottfried E, Noessner E. Tumor lactic acidosis suppresses CTL function by inhibition of p38 and JNK/c-Jun activation. Int J Cancer. 2012;131(3):633–40. 21898391 10.1002/ijc.26410

[51] Calcinotto A, Filipazzi P, Grioni M, Iero M, De Milito A, Ricupito A, et al. Modulation of microenvironment acidity reverses anergy in human and murine tumor-infiltrating T lymphocytes. Cancer Res. 2012;72(11):2746–56. 22593198 10.1158/0008-5472.CAN-11-1272

[52] Navarrete-Bernal MGC, Cervantes-Badillo MG, Martinez-Herrera JF, Lara-Torres CO, Gerson-Cwilich R, Zentella-Dehesa A, et al. Biological Landscape of Triple Negative Breast Cancers Expressing CTLA-4. Front Oncol. 2020;10:1206. 32850353 10.3389/fonc.2020.01206 PMC7419680

[53] Boudreau A, Purkey HE, Hitz A, Robarge K, Peterson D, Labadie S, et al. Metabolic plasticity underpins innate and acquired resistance to LDHA inhibition. Nat Chem Biol. 2016;12(10):779–86. 27479743 10.1038/nchembio.2143

[54] Yeung C, Gibson AE, Issaq SH, Oshima N, Baumgart JT, Edessa LD, et al. Targeting Glycolysis through Inhibition of Lactate Dehydrogenase Impairs Tumor Growth in Preclinical Models of Ewing Sarcoma. Cancer Res. 2019;79(19):5060–73. 31431459 10.1158/0008-5472.CAN-19-0217 PMC6774872

[55] Wolchok JD, Chiarion-Sileni V, Gonzalez R, Rutkowski P, Grob JJ, Cowey CL, et al. Overall Survival with Combined Nivolumab and Ipilimumab in Advanced Melanoma. N Engl J Med. 2017;377(14):1345–56. 28889792 10.1056/NEJMoa1709684 PMC5706778

[56] Reck M, Schenker M, Lee KH, Provencio M, Nishio M, Lesniewski-Kmak K, et al. Nivolumab plus ipilimumab versus chemotherapy as first-line treatment in advanced non-small-cell lung cancer with high tumour mutational burden: patient-reported outcomes results from the randomised, open-label, phase III CheckMate 227 trial. Eur J Cancer. 2019;116:137–47. 31195357 10.1016/j.ejca.2019.05.008

[57] Grimm MO, Esteban E, Barthelemy P, Schmidinger M, Busch J, Valderrama BP, et al. Tailored immunotherapy approach with nivolumab with or without nivolumab plus ipilimumab as immunotherapeutic boost in patients with metastatic renal cell carcinoma (TITAN-RCC): a multicentre, single-arm, phase 2 trial. Lancet Oncol. 2023;24(11):1252–65. 37844597 10.1016/S1470-2045(23)00449-7

[58] Gao J, Shi LZ, Zhao H, Chen J, Xiong L, He Q, et al. Loss of IFN-gamma Pathway Genes in Tumor Cells as a Mechanism of Resistance to Anti-CTLA-4 Therapy. Cell. 2016;167(2):397-404 e9. 27667683 10.1016/j.cell.2016.08.069 PMC5088716

[59] Carlino F, Diana A, Piccolo A, Ventriglia A, Bruno V, De Santo I, et al. Immune-Based Therapy in Triple-Negative Breast Cancer: From Molecular Biology to Clinical Practice. Cancers (Basel). 2022;14(9):2102. 10.3390/cancers14092102 PMC9103968 35565233

[60] Van Heertum RL, Greenstein EA, Tikofsky RS. 2-deoxy-fluorglucose-positron emission tomography imaging of the brain: current clinical applications with emphasis on the dementias. Semin Nucl Med. 2004;34(4):300–12. 15493007 10.1053/j.semnuclmed.2004.03.003

