# Supplementary material for: NAT10/ac4C/JunB facilitates TNBC malignant progression and immunosuppression by driving glycolysis addiction
Source: J Exp Clin Cancer Res. 2024 Oct 4;43:278. doi: 10.1186/s13046-024-03200-x (PMC11451012; doi:10.1186/s13046-024-03200-x)
Supplement: Supplementary file 5 — Supplementary Material 5: Supplementary Data 5. CyTOF antibody information [file 13046_2024_3200_MOESM5_ESM.docx]

| **List** | **Label** | **marker** | **clone** | **dilution** | **品牌** |
| --- | --- | --- | --- | --- | --- |
| **1** | **89Y** | **CD45** | **30-F11** | **400** | Biolegend |
| **2** | **115ln** | **CD3ε** | **145-2C11** | **50** | Biolegend |
| **3** | **141Pr** | **CD49b(pan-NK cells)** | **DX5** | **100** | Biolegend |
| **4** | **142Nd** | **MHC II(I-A/I-E)** | **M5/114.15.2** | **400** | Biolegend |
| **5** | **143Nd** | **IL-17A** | **TC11-18H10.1** | **100** | Biolegend |
| **6** | **144Nd** | **CX3CR1** | **SA011F11** | **100** | Biolegend |
| **7** | **145Nd** | **CD152(CTLA-4)** | **UC10-4B9** | **100** | Biolegend |
| **8** | **146Nd** | **CD206(MMR)** | **C068C2** | **50** | Biolegend |
| **9** | **147Sm** | **Ly-6G** | **1A8** | **800** | Biolegend |
| **10** | **148Nd** | **Ly-6C** | **HK1.4** | **800** | Biolegend |
| **11** | **149Sm** | **CD19** | **6D5** | **200** | Biolegend |
| **12** | **150Nd** | **IL-10** | **JES5-16E3** | **50** | Biolegend |
| **13** | **151Eu** | **CD44** | **IM7** | **100** | Biolegend |
| **14** | **152Sm** | **CD11c** | **N418** | **100** | Biolegend |
| **15** | **153Eu** | **IL-4** | **11B11** | **50** | Biolegend |
| **16** | **154Sm** | **CD62L** | **MEL-14** | **200** | Biolegend |
| **17** | **155Gd** | **CD103** | **2E7** | **200** | Biolegend |
| **18** | **156Gd** | **IL-33R(IL1RL1,ST2)** | **DIH9** | **100** | Biolegend |
| **19** | **157Gd** | **CD25** | **3C7** | **100** | Biolegend |
| **20** | **158Gd** | **CD45R(B220)** | **RA3-6B2** | **200** | Biolegend |
| **21** | **159Tb** | **F4/80** | **Cl:A3-1** | **200** | BioRAD |
| **22** | **160Gd** | **CD274(B7-H1,PD-L1)** | **10F.9G2** | **200** | Biolegend |
| **23** | **161Dy** | **CD279(PD-1)** | **29F.1A12** | **50** | Biolegend |
| **24** | **162Dy** | **CD183(CXCR3)** | **CXCR3-173** | **100** | Biolegend |
| **25** | **163Dy** | **CD185(CXCR5)** | **L138D7** | **100** | Biolegend |
| **26** | **164Dy** | **CD86** | **GL-1** | **200** | Biolegend |
| **27** | **165Ho** | **IFN-γ** | **XMG1.2** | **100** | Bio-Xcell |
| **28** | **166Er** | **CD192(CCR2)** | **475301** | **50** | RD |
| **29** | **167Er** | **TCR β chain** | **H57-597** | **200** | Biolegend |
| **30** | **168Er** | **FOXP3** | **FJK-16s** | **100** | ebioscience |
| **31** | **169Tm** | **CD163** | **S15049I** | **100** | Biolegend |
| **32** | **170Er** | **iNOS** | **CXNFT** | **50** | ebioscience |
| **33** | **171Yb** | **CD69** | **H1.2F3** | **100** | Biolegend |
| **34** | **172Yb** | **CD127(IL-7Rα)** | **A7R34** | **50** | Biolegend |
| **35** | **173Yb** | **Granzyme B** | **GB11** | **100** | **Fluidigm** |
| **36** | **174Yb** | **CD196(CCR6)** | **29-2L17** | **50** | BD |
| **37** | **175Lu** | **CD68** | **FA-11** | **100** | Biolegend |
| **38** | **176Yb** | **TNF-α** | **MP6-XT22** | **100** | Biolegend |
| **39** | **197Au** | **CD4** | **RM4-5** | **800** | Biolegend |
| **40** | **198pt** | **CD8a** | **53-6.7** | **400** | Biolegend |
| **41** | **209Bi** | **CD11b** | **M1/70** | **800** | Biolegend |
