# Supplementary material for: NAT10/ac4C/JunB facilitates TNBC malignant progression and immunosuppression by driving glycolysis addiction
Source: J Exp Clin Cancer Res. 2024 Oct 4;43:278. doi: 10.1186/s13046-024-03200-x (PMC11451012; doi:10.1186/s13046-024-03200-x)

Supplementary Figure S1

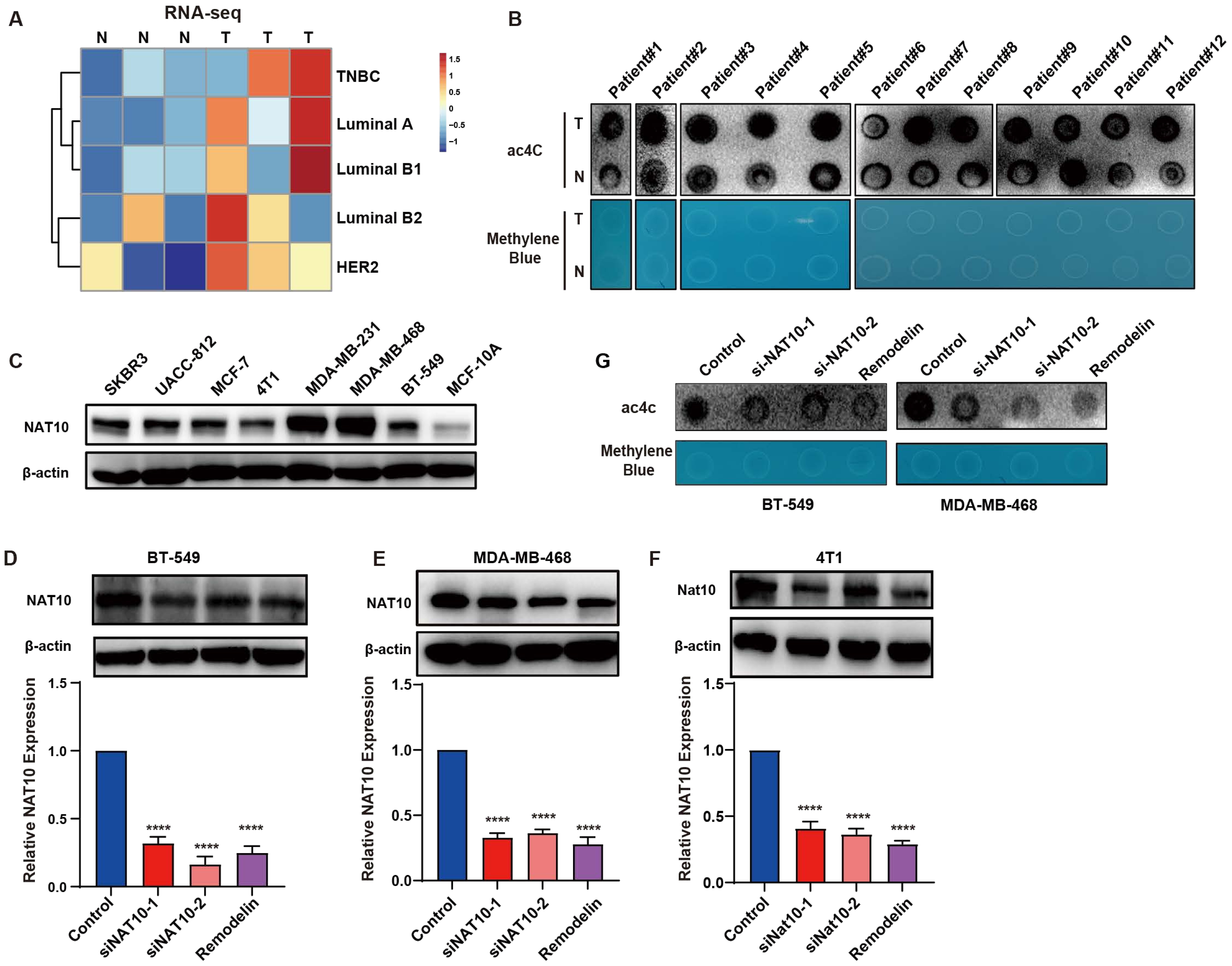

Supplementary Figure S2

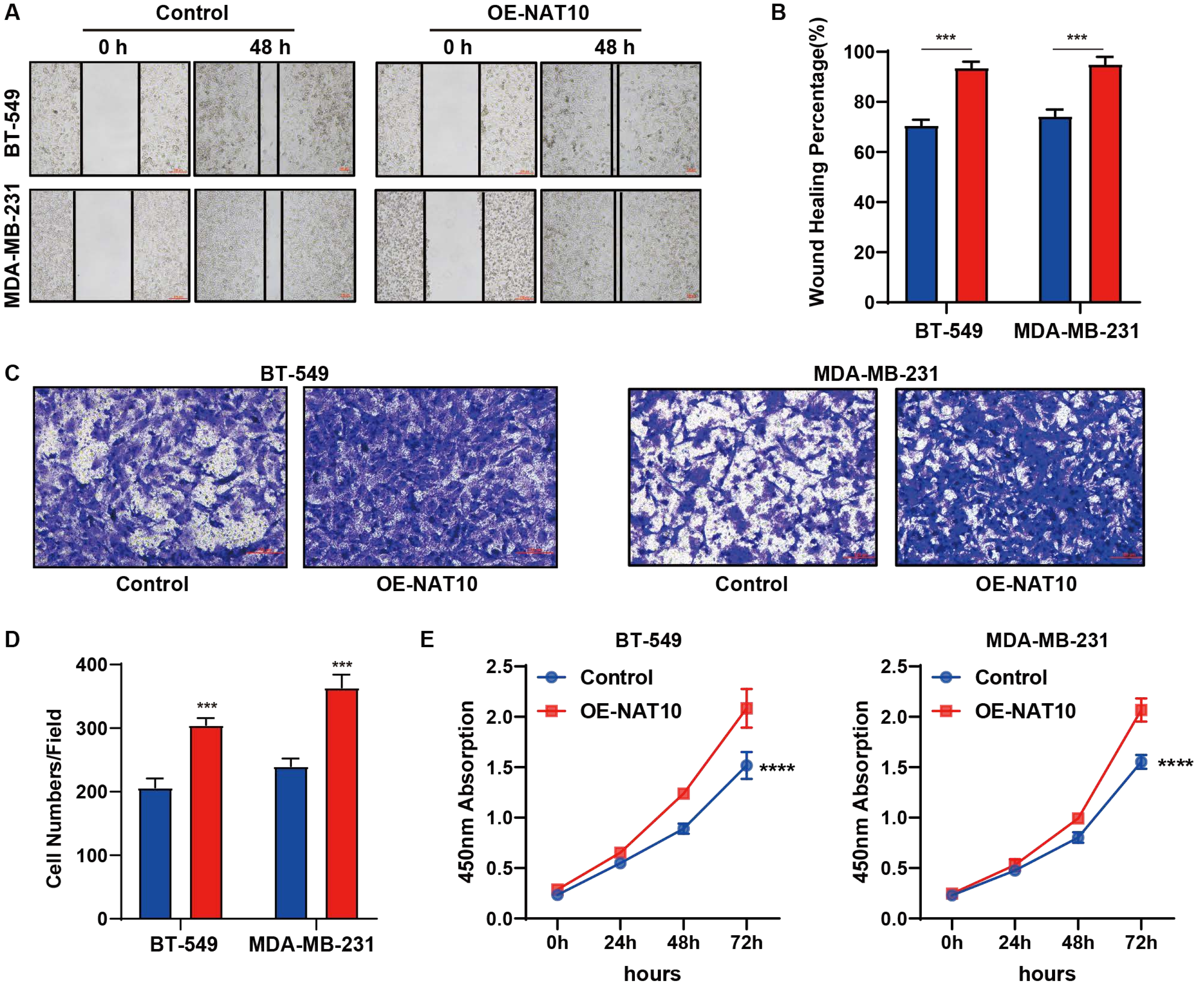

Supplementary Figure S3

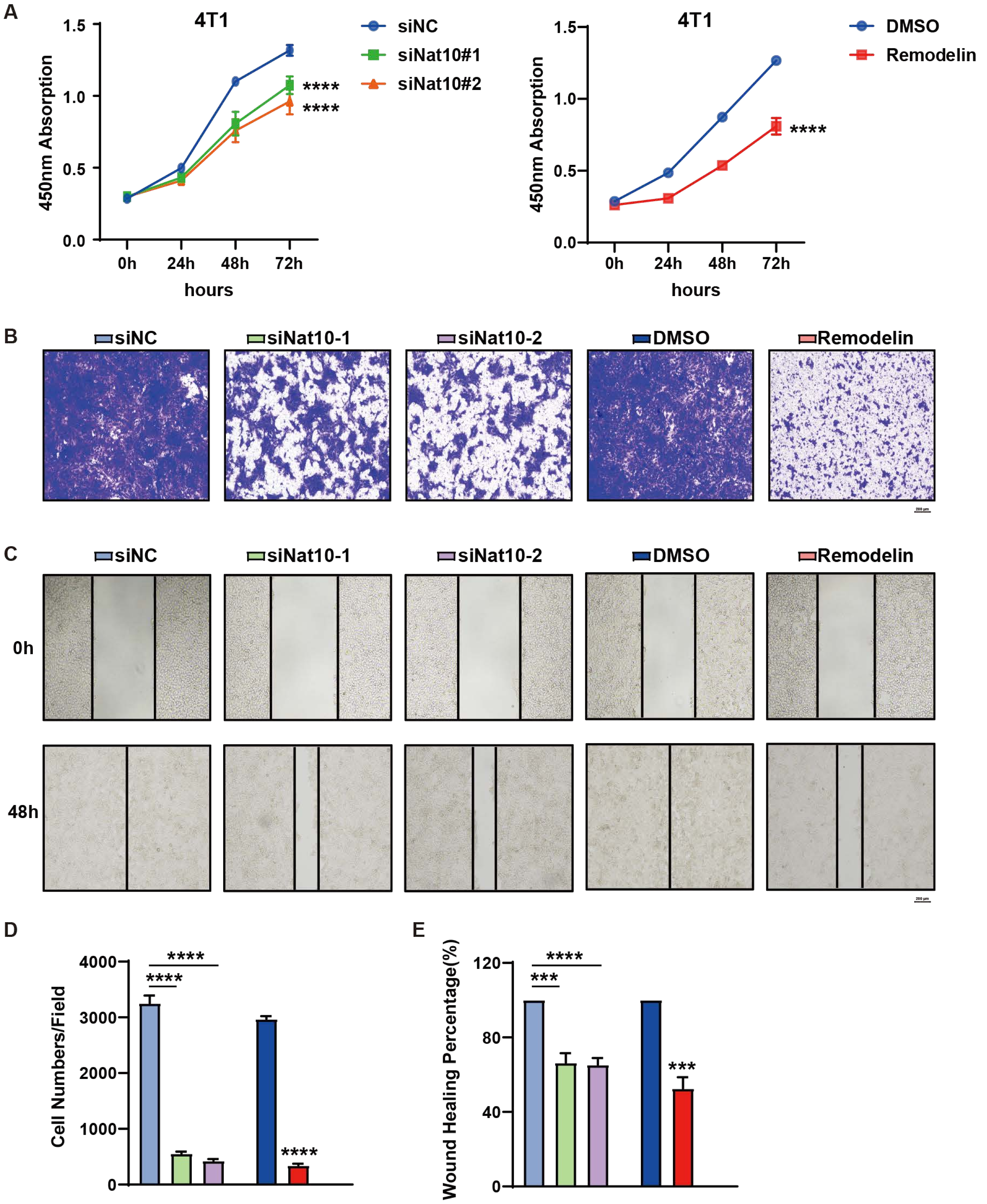

Supplementary Figure S4

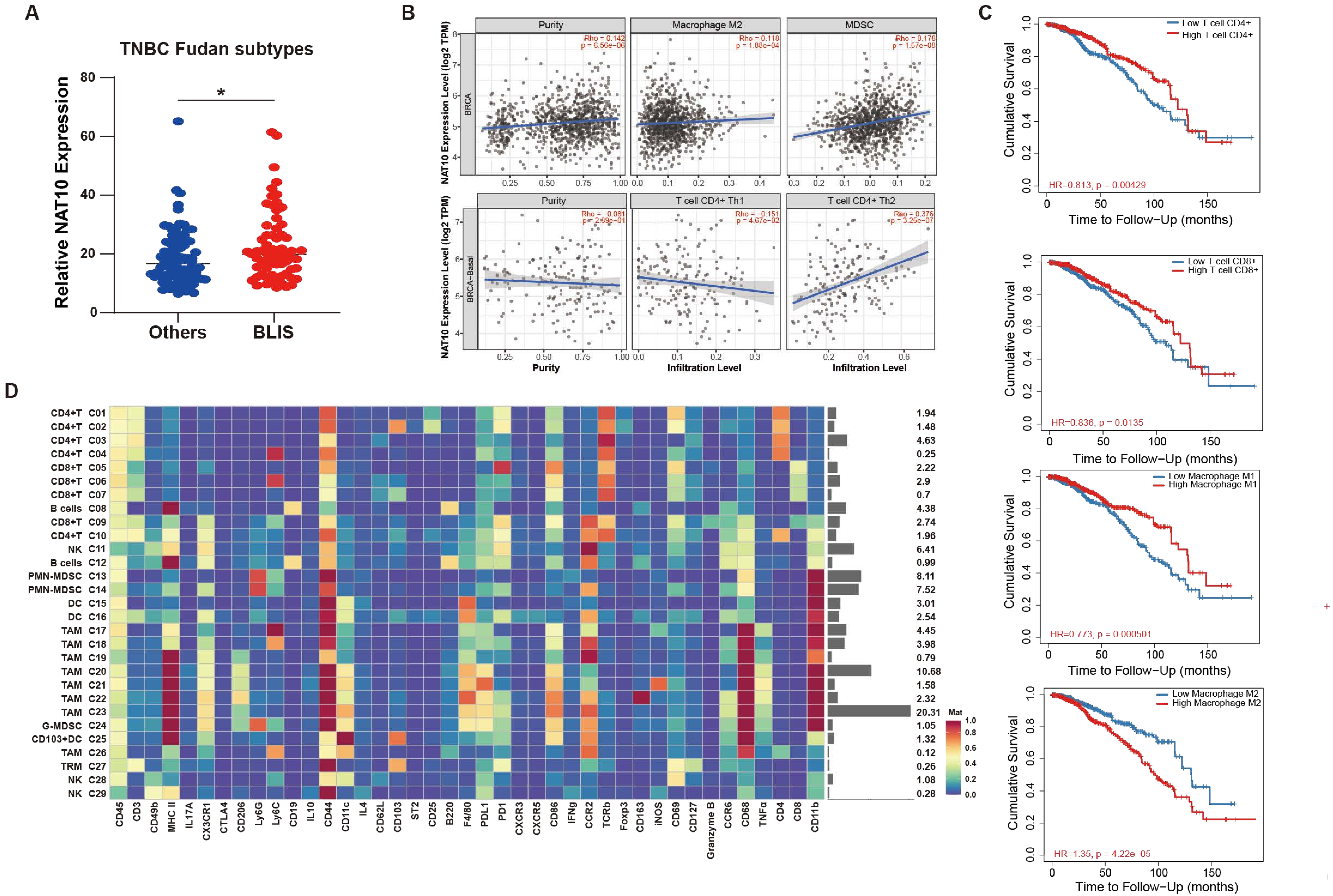

Supplementary Fig. S5

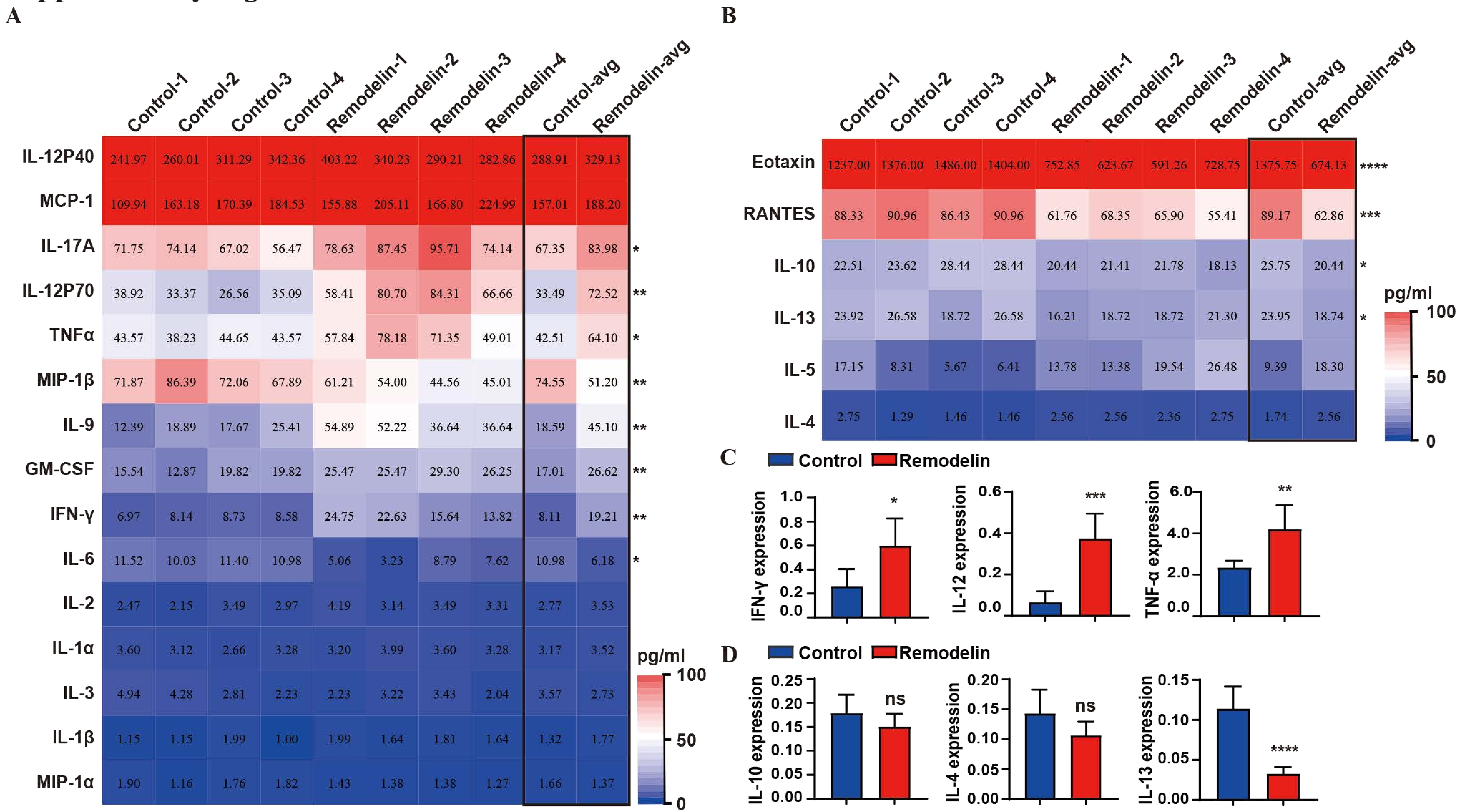

Supplementary Figure S6

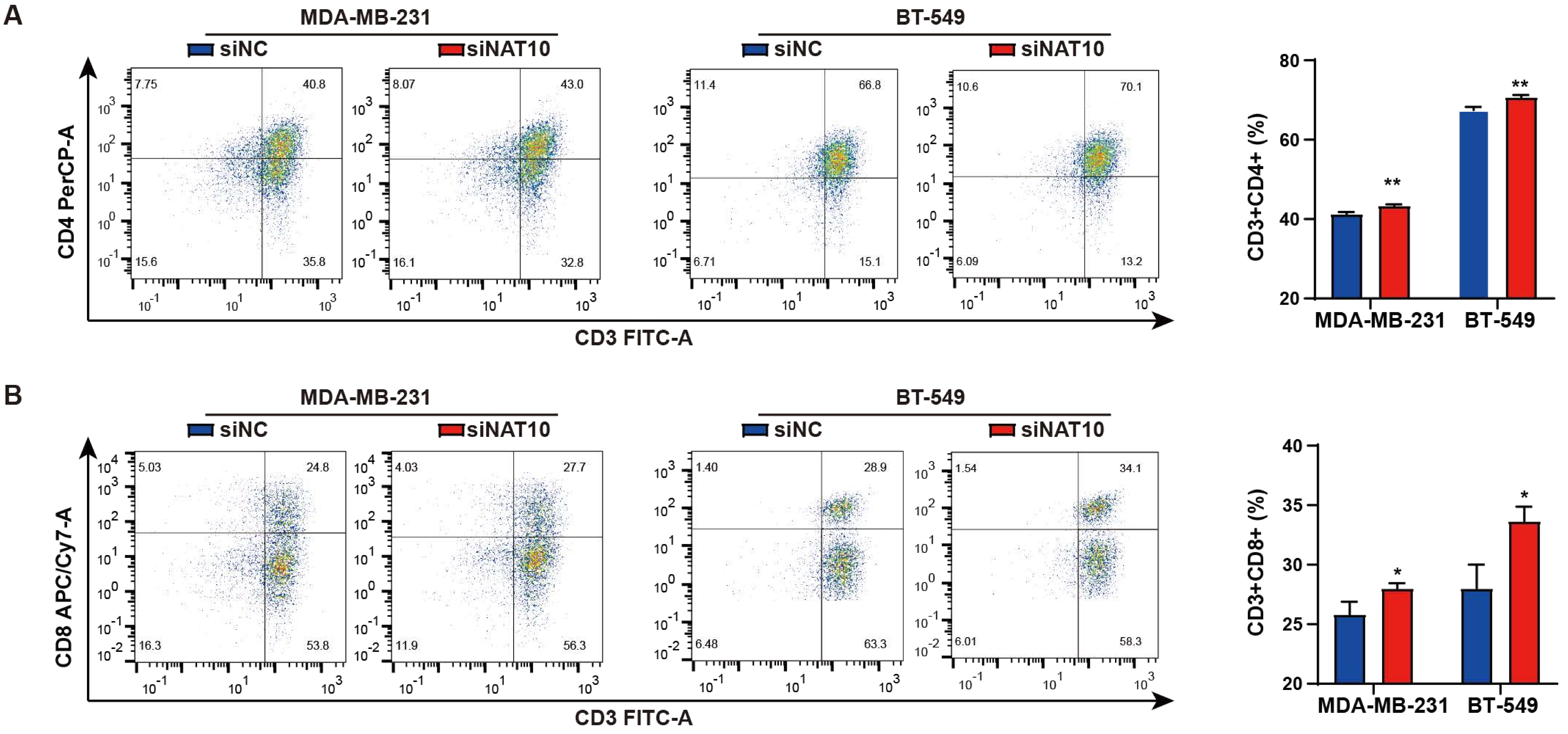

Supplementary Figure S7

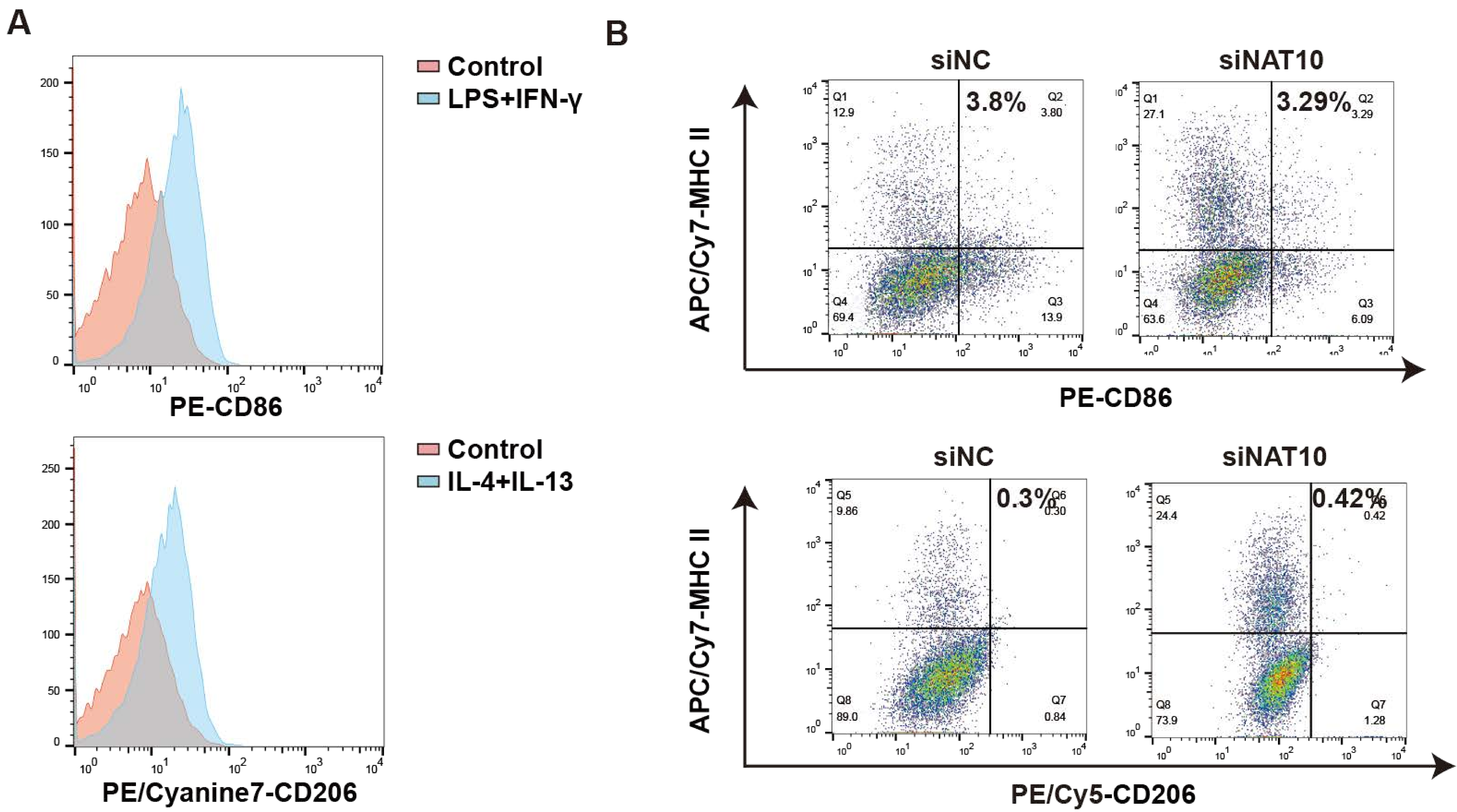

Supplementary Figure 8

A

ID	Sequence	Start	End	Score thresholds: 0.4
JunB 3726	CTCTCTCTACACGACTACAAACTCCTGAAACCGAGCCTGGCGGTCAACCTGG<br>CCGACCCCTACCGGAGTCTCAAAGCGCCTGGGGCTCGCGGACCCGGCCCAGA<br>GGGCGGCGGTGGCGGCAGCTACTTTTCTGGTCAGGGCTCGGACACCGGCGCGT<br>CTCTCAAGCTCGCCTCTTCGGAGCTGGAACGCCTGATTGTCCCCAACAGCAA<br>CGGCGTGATCACGACGACGCCTACACCCCCGGGACAGTACTTTACCCCCGC<br>GGGGGTGGCAGCGGTGGAGGTGCAGGGGGCGCAGGGGGCGGCGTCACCGAGG<br>AGCAGGAGGGCTTCGCCGACGGCTTTGTCAAAGCCCTGGACGATCTGCACAA<br>GATGAACCACGTGACACCCCCCAACGTGTCCCTGGGCGCTACCGGGGGGC	279	293	0. 4255

Zhao W., Zhou Y., Cui Q. & Zhou Y. PACES: prediction of N4-acetylcytidine (ac4C) modification sites in mRNA. Sci Rep 9, 11112, doi:10.1038/s41598-019-47594-7 (2019).

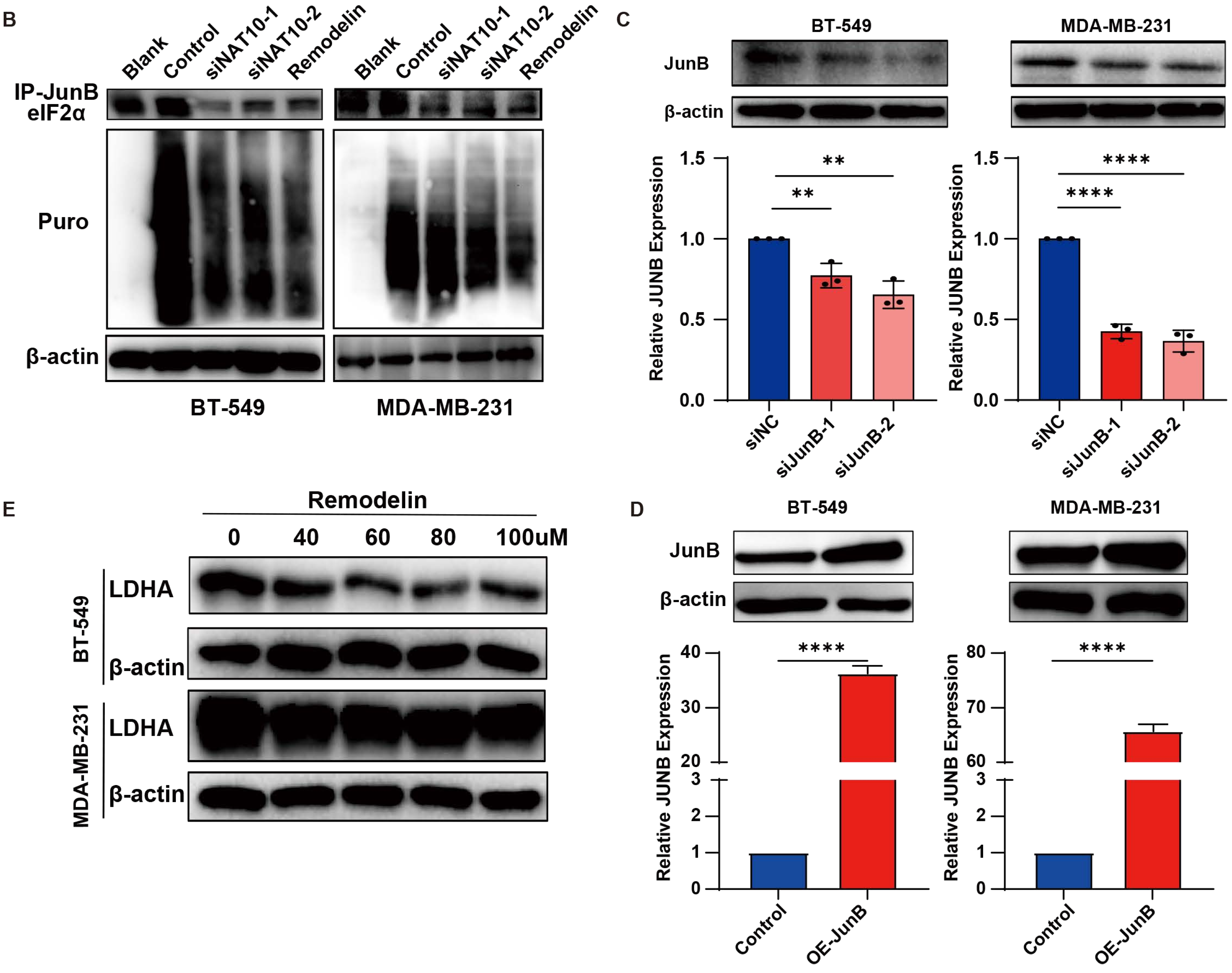

Supplementary Figure S9

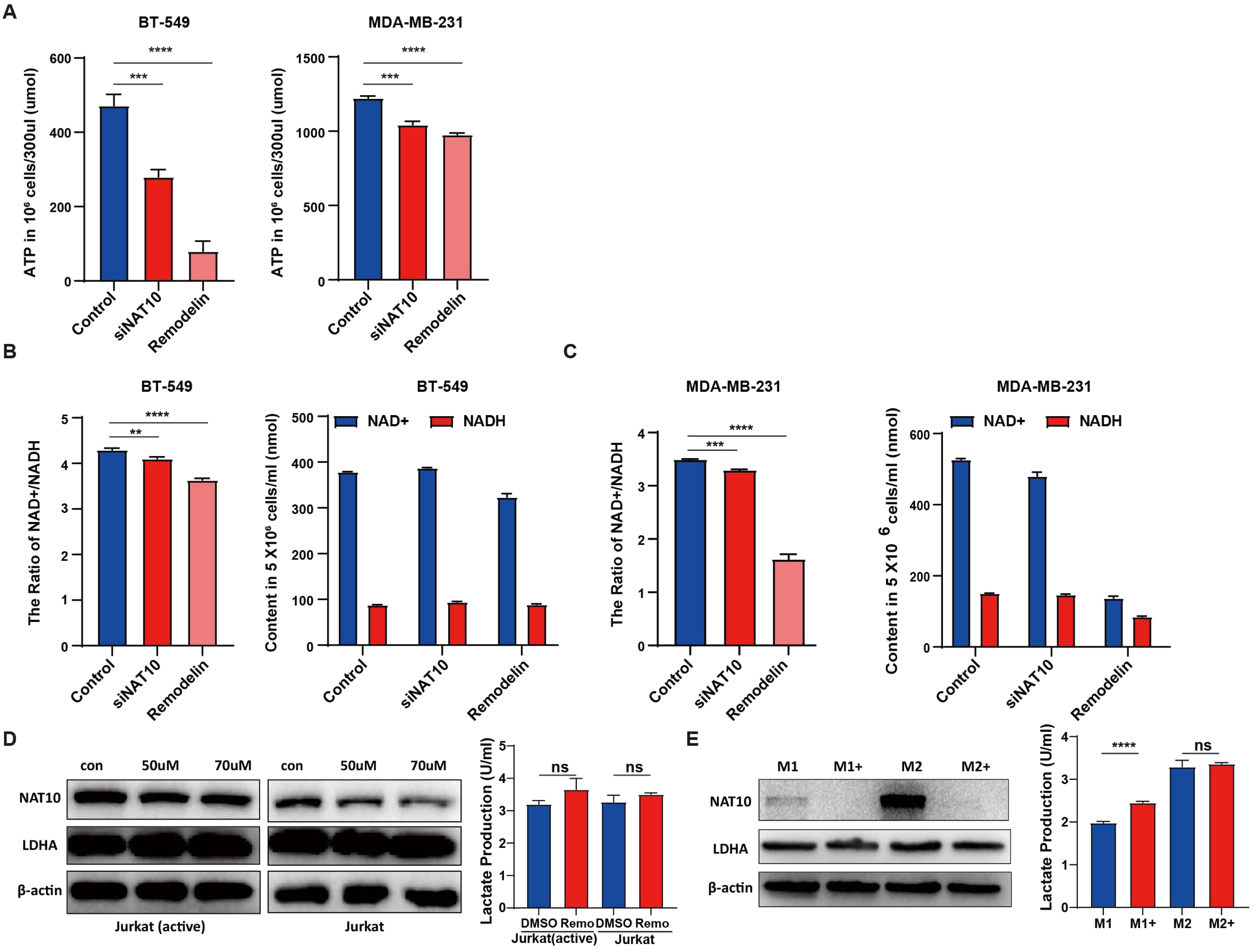

Supplementary Figure S10

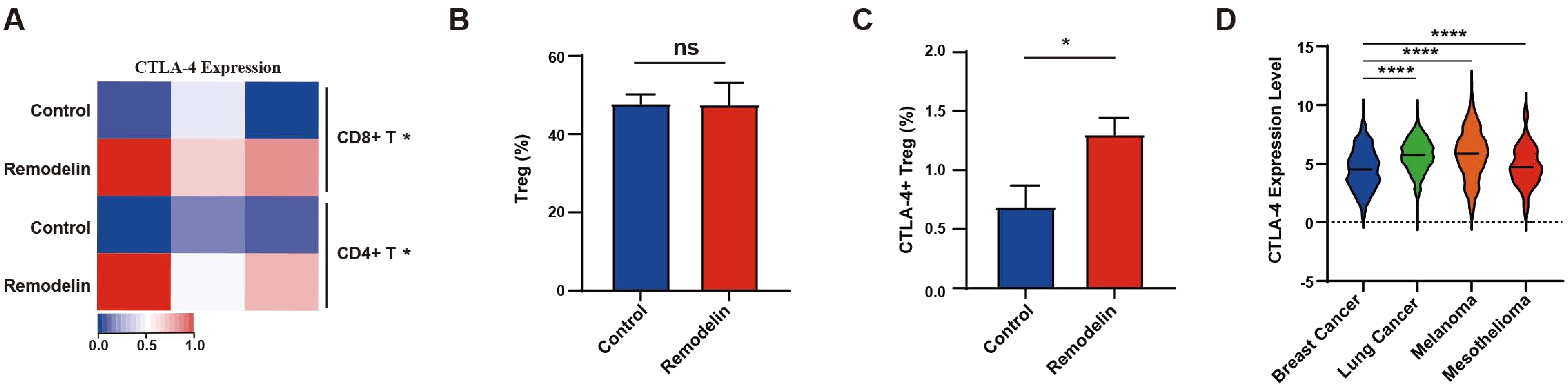

Supplementary Figure S11

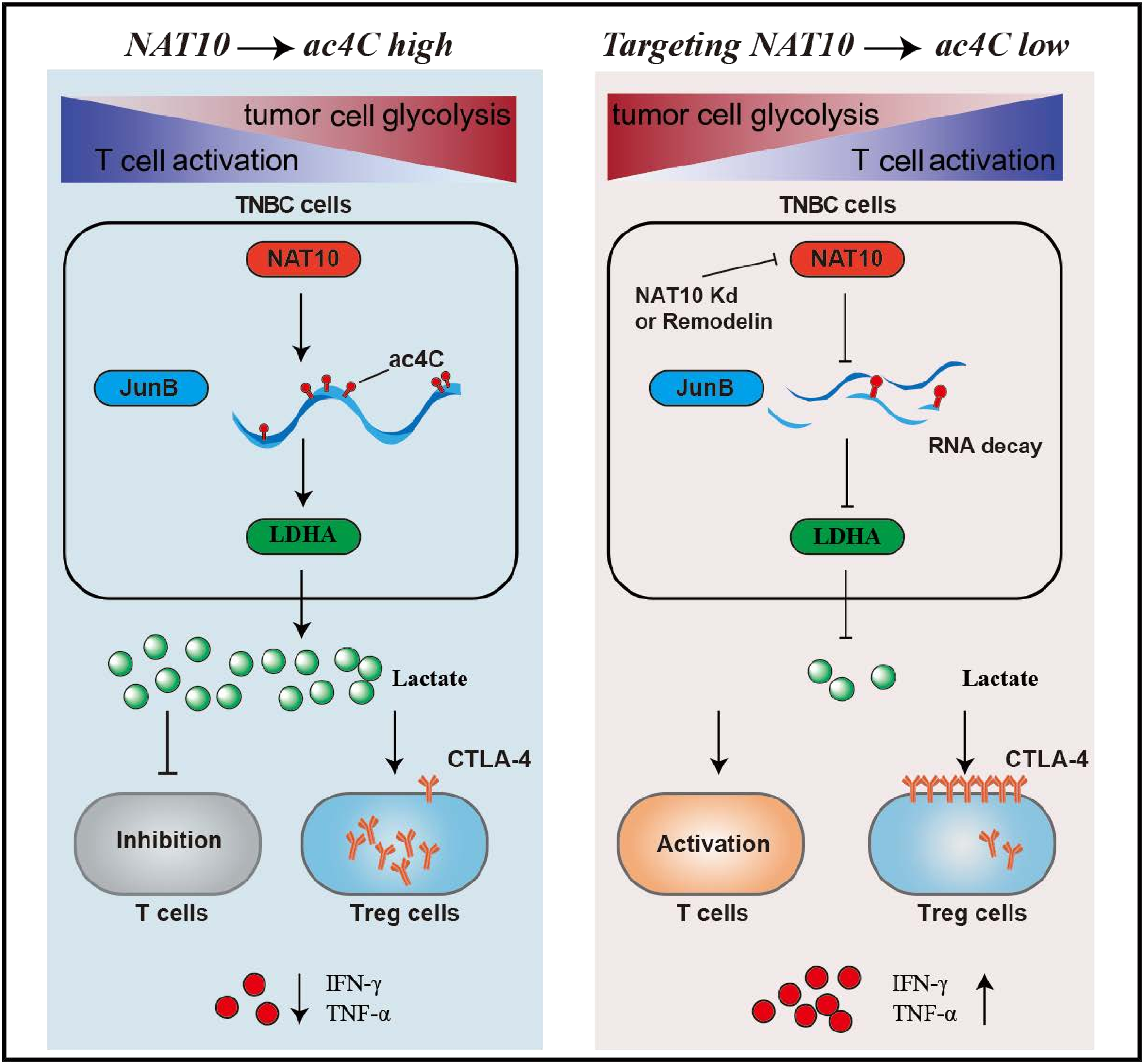

Supplement: Supplementary file 7 — Supplementary Material 7: Supplementary Fig. S1. Ac4C level in TNBC tissues and efficiency of targeting NAT10. (A) Heat map showed the expression of NAT10 in different molecular types. (B)The ac4C level in TNBC tissues was determined by dot blot. (C) The NAT10 expression in different breast cancer cell lines. (D-F) Western blot and qRT-PCR measured NAT10 expression by using siRNA and remodelin. (G) Dot-blot quantification of ac4C abundance in mRNA transcripts. Ac4C dot blot assay was performed with methylene blue (MB) as a loading control. The data are shown as the means ± SDs; ****P < 0.0001. Supplementary Fig. S2. NAT10 facilitates the TNBC tumorigenesis. (A-D) NAT10 overexpression promotes the TNBC cells invasion and migration. (E) NAT10 overexpression promotes the TNBC cells proliferation. The data are shown as the means ± SDs; ****P < 0.0001. Supplementary Fig. S3. NAT10 facilitates the 4T1 cell development. (A) The loss of NAT10 inhibits the 4T1 proliferation. (B-E) The loss of NAT10 inhibits the 4T1 invasion and migration. The data are shown as the means ± SDs; ***P < 0.001, ****P < 0.0001. Supplementary Fig. S4. NAT10 function is required for immunosuppressive TME in TNBC. (A) NAT10 expression in BLIS and non-immunosuppressive subtype (Others), generated from TCGA database (B) Correlation of NAT10 expression with immune infiltration levels in breast cancer based on the TIMER2 analysis. (C) Kaplan-Meier survival curves comparing immune cell infiltration of breast cancer in TIMER2. (D) Heatmap of phenograph clusters of CD45+ cells. The relative expression levels of markers across cells were shown and sorted by cell type. The data are shown as the means ± SDs; *P < 0.05. Supplementary Fig. S5. Targeting NAT10 increases cytokines that could activate T cell and macrophages. (A-B) Heatmap showing serum cytokine concentration of remodeling-treated mice bearing 4T1 tumor compared with control. (C-D) Mice tissues were used to detect the mRNA expression of cytokines. [file 13046_2024_3200_MOESM7_ESM.pdf]
